# Early exploitation of Neapolitan pozzolan (*pulvis puteolana*) in the Roman theatre of Aquileia, Northern Italy

**DOI:** 10.1038/s41598-023-30692-y

**Published:** 2023-03-13

**Authors:** Simone Dilaria, Michele Secco, Andrea R. Ghiotto, Guido Furlan, Tommaso Giovanardi, Federico Zorzi, Jacopo Bonetto

**Affiliations:** 1 Department of Cultural Heritage (DBC), University of Padova, Piazza Capitaniato 7, 35139 Padua, Italy; 2 Inter, Departmental Research Centre for the Study of Cement Materials and Hydraulic Binders (CIRCe), University of Padova, Via Giovanni Gradenigo 6, 35131 Padua, Italy; 3 Centre for Urban Network Evolutions—UrbNet, School of Culture and Society, Aarhus University, Moesgård Allé 20, 4230-223, 8270 Højbjerg, Denmark; 4 Department of Chemical and Geological Sciences, University of Modena and Reggio Emilia, Via Campi 103, 41125 Modena, Italy; 5 Analysis Center and Certification Services (CEASC), University of Padova, Via Jappelli 1/A, 35121 Padua, Italy

**Keywords:** Archaeology, Geochemistry, Composites, Scanning electron microscopy, Transmission light microscopy, Characterization and analytical techniques

## Abstract

The paper reports the results of the analyses on mortar-based materials from the Roman theatre of Aquileia (Friuli Venezia Giulia, Northern Italy), recently dated between the mid-1st Century BCE and the mid-1st Century CE. Samples were characterized by Polarized Light Microscopy on thin sections (PLM), Scanning Electron Microscopy with Energy Dispersive Spectroscopy (SEM–EDS) and Quantitative Phase Analysis by X-Ray Powder Diffraction (QPA-XRPD). Pyroclastic aggregates (mainly pumices and scattered tuffs), incompatible with the regional geology, were found in two samples from the preparation layers of the ground floor of the building. Their provenance was determined by means of QPA-XRPD, SEM–EDS, X-Ray Fluorescence (XRF) and Laser-Ablation Inductively-Coupled-Plasma Mass-Spectrometry (LA-ICP-MS). Mineralogical and geochemical analyses demonstrated their provenance from the Bay of Naples, thus recognizing them as *pulvis puteolana*, a type of pozzolanic aggregate outcropping around the modern town of Pozzuoli and prescribed by Vitruvius (*De Architectura*, 2.6.1) in mortar-based materials to strengthen masonries and produce hydraulic concrete for harbor piers. This evidence represents the oldest analytically-established case of *pulvis puteolana* exploitation in Northern Italy up to now, and an early use of the material out of Campania adapted for civil constructions in a non-strictly maritime-related environment. Indeed, the theatre was built in the low-lying Aquileia’s deltaic plain, prone to water infiltrations that are typical in lagoon-like environments. The data highlight the craftsmen’s resilience in adapting and reinterpreting the traditional use of the Neapolitan volcanic materials to deal with the geomorphological challenges of Aquileia’s lowland.

## Introduction

Volcanic pozzolans are different types of highly amorphous, poorly coherent rocks rich in reactive silica and alumina having mainly a pyroclastic origin. In the production of mortar-based materials, once mixed with water they interact with the slaked aerial lime (portlandite), inducing the dissolution of the aluminosilicate phases to generate a series of calcium-based reaction products (calcium silicate hydrate C–S–H, calcium aluminate hydrate C–A–H and calcium aluminosilicate hydrate C–A–S–H) structurally affine to the mineral phases occurring in natural hydraulic lime and modern cement^1–6^.

The word "pozzolanic" originates from the Latin term *puteolanus*, credited by Pliny the Elder (*Naturalis Historia*, 16.202; 35.166) to a particular natural ash outcropping close to the modern city of Pozzuoli in the Bay of Naples, and mentioned for the very first time (1st Century BCE) by Vitruvius as a *pulvis* (powder) that can be sourced between *Baiae* and the area around Mount Vesuvius (*De Architectura*, 2.6.1–2; 5.12.2). Both authors considered the *pulvis puteolana* a prodigious powder to be used in the manufacture of mortar-based materials, to strengthen masonry and to produce hydraulic concrete for harbor piers.

According to modern geology, this material corresponds to the pyroclastic flows and fallout deposits (i.e. pumices and tuffs) of the volcanic units located around the Bay of Naples, with specific reference to the Quaternary eruptions of the Phlegraean Fields and those of Somma-Vesuvius pre-dating 79 CE^7–12^.

*Pulvis puteolana* is not the only volcanic pozzolan mentioned in the treatises of the Latin authors. Vitruvius (*De Architectura*, 2.4.1) is the first author mentioning the *harenae fossiciae* as quarry sands, having different colors (*rubra*, *nigra* and *cana*), that were employed in mortar-based materials to strengthen masonry. The *harenae fossiciae* are generally identified^8,13,14^ with the volcanic ashes of the Middle Pleistocene eruptions of volcanoes of the Latium Province (Vulsini, Vico, Monti Sabatini and Colli Albani).

Beyond the “traditional” volcanic pozzolans reported by Roman authors, archaeological evidence demonstrated that further volcanic products (i.e. lavas, obsidians, perlites) were exploited in the provinces of the Empire to produce hydraulic, stiff and durable mortar-based materials^15–20^.

However, the circulation of these “alternative” pozzolans has always remained intra-regional and essentially circumscribed to the sites near the sourcing quarries. This is also the case of the *harenae fossiciae*, that were exploited since the Middle-Late Republican age to produce mortar-based materials in Rome^9,13,14,21–26^ and in the sites around the city^8,26–28^.

As demonstrated by recent geoarchaeological research, only *pulvis puteolana* was broadly traded in the Mediterranean. It even reached the Levantine coasts, as evidenced by the presence of Phlegraean pumices and tuffs in the *opus caementicium* piers of the port of *Caesarea Maritima*, commissioned by King Herod between 23 and 15 BCE^7,29^. Together with the Phlegraean pozzolans, the Somma-Vesuvius volcanic products were massively exported too, as confirmed by the presence of pyroclastic aggregates displaying the geochemical fingerprint of Somma-Vesuvius products in the piers of the port of Chersonisos in Crete^7^.

The spread of Neapolitan pozzolans in the provinces of the Roman empire grew in a short timeframe. After the first experimentations in the construction of *opus caementicium* fish tanks in Tyrrhenian maritime villas of rich senators and contractors of the late Republican Age (late 2nd–1st Century BCE)^7,10,30^, this product gained monopoly in the markets within a few decades as an excellent raw material for the manufacture of durable hydraulic mortars and concretes.

The reason for this massive spread is probably related to trade logistics: the outcrops are located close to the coast of the Bay of Naples, where the great harbors of the Roman Era such as those of Puteoli, Baia and Miseno^31^ were established. These factors played a key role in the trade of the material, which travelled the sea as ship ballasts, together with handbooks and craftsmen, during Rome's rapid expansion throughout the Mediterranean Sea^7,9^.

The materials also reached the coasts of the Adriatic Sea, where its early employment far from the Bay of Naples was documented in the *opus caementicium* structures of the port of *Egnatia*^7,30^, dated at the time of the war between Octavianus and Marcus Antonius^32^. This was an extraordinary circumstance at the time, as the infrastructure was probably designed by the engineers of Octavian's army to be a military harbor in the Adriatic and a bridgehead to Actio.

Other verified instances of *pulvis puteolana* utilization were documented by the analysis of the *opus caementicium* infrastructures of massive Imperial harbors from Italy, Turkey and Egypt^7,10^. Currently, research only suggests the selective employment of the Neapolitan volcanic materials for high-profile maritime civil constructions, due to its preferential focus on Roman harbor infrastructures. In fact, in the sites around the Bay of Naples, the local volcanic pozzolan was also employed in the construction of public and private overground buildings, at least from the 3rd–2nd Century BCE onwards^33–37^.

In this paper, the results of the analyses on several mortar-based materials collected from the theatre of Aquileia (Friuli Venezia Giulia, Northern Italy) are reported. Pyroclastic aggregates (mainly pumices) were found in two samples, coming from the preparation layers of the floors of the *orchestra* and *hyposcaenium*. Petro-mineralogical and geochemical analyses demonstrated that these volcanic pozzolans were sourced in the Bay of Naples. Cross-referencing the analytical data with the dating of the building indicates that this is the oldest analytically established case of employment of *pulvis puteolana* in Northern Italy up to date; it represents also an early utilization of the material adapted for civil constructions in non-strictly maritime-related environments far from the Bay of Naples.

## The Roman theatre of Aquileia

### The building and its chronology

Aquileia was one of the main Roman towns of the ancient *Cisalpina* region (roughly corresponding to current Northern Italy). Established in 181 BCE in an inland area of the Friuli lowland, approximately 10 km from the coast of the northern Adriatic Sea, the colony represented a bridgehead of the Roman culture to the north of the Italian peninsula. In later centuries, Aquileia developed into a flourishing urban center enriched with monumental buildings and prestigious private houses. In the late fourth century CE Ausonius (*Ordo urbium nobilium*, IX) mentioned it as one of the largest cities in the Roman world^38^.

In the past decades, the existence and approximate location of the Roman theatre within the urban plan of Aquileia was suggested by archaeologist Luisa Bertacchi^39^. Recent archaeological activities, carried out since 2015 by the University of Padova^40–44^, revealed its exact location, not far from the urban *forum* and immediately outside the Republican walls. The excavations defined the planimetric articulation, dimensions and building techniques of the theatre, which has a diameter of approximately 95 m. It is therefore one of the largest Roman theatres in *Cisalpina*, together with the neighboring theatres of Pola (Large Theatre), Padova and Verona^42^.

**Figure 1:**
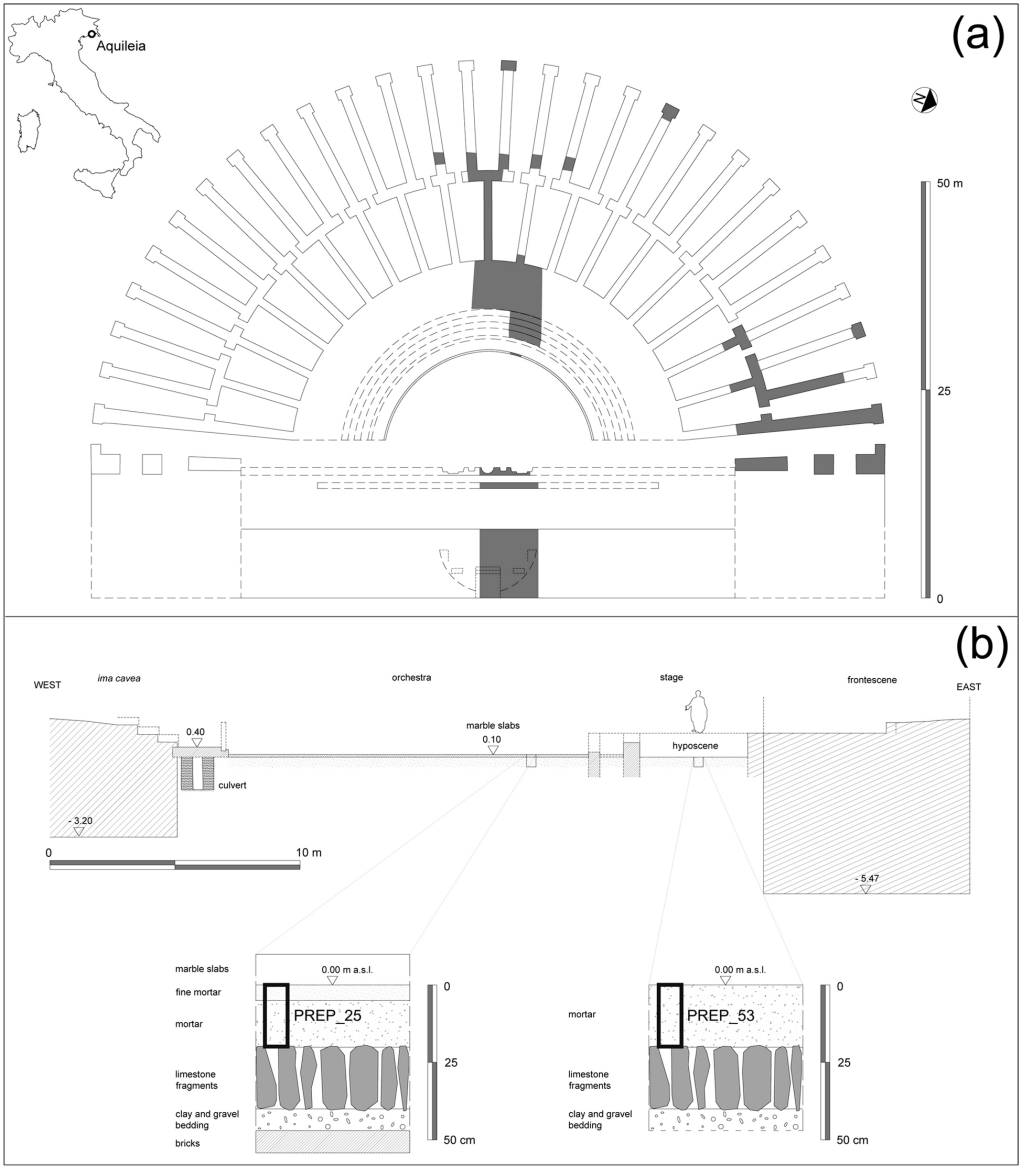
The Roman theatre in Aquileia. (**a**) Reconstructive plan of the building with indication of the excavated sectors (in dark grey); (**b**) Reconstructive cross section of the theatre, from the *ima cavea* to the *scaenae frons* wall, with stratigraphic sketches of the floor preparations of the *orchestra* and *hyposcaenium*.

The structure was built in a low-lying land possibly affected by water infiltrations, an issue involving the whole Aquileia’s floodplain in antiquity^45,46^. Such factor required an adequate consolidation of its foundations. In fact, the curvilinear sector of the theatre (*cavea*, Fig. 1a), intended to accommodate the public, was placed over a foundational *opus caementicium* substructure divided into three concentric sectors, corresponding in elevation to the tiers of steps of the *summa*, *media* and *ima cavea*. The outer and middle sub-structural sectors are marked by a regular pattern of radial walls, separated by an intermediate curvilinear wall. These structures are built with a core in *opus caementicium* and a *paramentum* of small limestone blocks, with occasional rows of bricks. Only the inner sector (*ima cavea*) is composed of a solid structure. The outer radial walls externally end with pillars made of limestone blocks, marking the openings of the building; the stairs leading to the tiers of seats were placed at regular intervals. The main entrances consist in two long corridors (*aditus maximi*), located at the ends of the *cavea*, leading to the semi-circular area of the *orchestra*. It has a diameter of around 29.5 m, and it shows traces of the original flooring in white marble slabs.

In front of the *orchestra*, the excavations brought to light the low front wall of the stage (*frons pulpiti*). The stage floor, presumably in wooden planking (not preserved), was placed between this structure and the *scaenae frons*, and it covered the *hyposcaenium* beneath it.

Behind the stage, there was the monumental *scaenae frons* wall, around 8.2 m thick. In the middle of this structure, traces of a large niche, around 12.0 m wide, are still recognizable; this niche framed the main door through which the actors entered the scene (*valva regia*).

Finally, a complex system of culverts under the floors allowed the drainage of wastewaters out of the building.

The overall layout of the building, its architectural decoration^43^, and the preliminary study of the finds (including ^14^C dated organic remains from foundational layers, see Supplementary Figure 1) suggest that the theatre was built between the mid-1st Century BCE and the mid-1st Century CE, most probably before 30 CE.

### The ground floor preparation techniques

Except for culverts, occasionally emptied and maintained, the *orchestra* and the *hyposcaenium* were placed at the two lowest heights of the theatre^43,44^.

The floor bedding sequences of the two sectors were investigated through two small test pits, revealing two very similar stratigraphical sequences (Fig. 1b), examined for about 0.45 m from the surface of the two floors. In the *orchestra*, the deepest documented layer consists of horizontal fragmented bricks (upper face at 0.4 m below the sea level); on this flat surface, a 7.5 cm thick layer composed of clay mixed with gravel was laid. The upper preparation layers of the floor match the Vitruvian prescriptions (*De Architectura*, 7.1.1–3). Indeed, the plastic coat formed a substrate for vertically fitting limestone fragments about 20.0 cm long (*statumen*). This technique maintained several empty spaces among the single elements, providing drainage against humidity. This preparatory layer was covered with a thick mortar-based screed, containing abundant sub-centimetric lithic and ceramic aggregates (*rudus*). Finally, this layer was covered by a 5.0 cm thick layer of slightly finer mortar (*nucleus*); the overall thickness of the screed was therefore about 20.0 cm. At this elevation (+ 0 m a.s.l.), the 10.0 cm thick white marble slabs constituting the floor of the *orchestra* were laid down.

A very similar sequence was revealed in the test pit excavated in the *hyposcaenium*, although it was not possible to ascertain the presence of the lowest bricks and the mortar screed did not display any internal stratification. The *hyposcaenium*, being a service space, was not accessible nor visible to the public; therefore, its mortar-based floor was not covered with stone slabs, as evidenced by the presence of nails and metal staples on its surface.

### Sampling and analysis

Samples of mortar-based materials were collected from different structural elements of the theatre (Supplementary Figure 2), namely:

- 11 samples from the *opus caementicium* sector of the *ima cavea* (PREF). The first pair comes from the exposed portion of this structure, while all other samples were selected at different depths from a drill cored in the foundational substructure of the *ima cavea*;
- 13 samples from the walls of the *cavea* and *scaenae frons*, respectively (WM);
- 2 samples from the preparatory layers of the *orchestra* and *hyposcaenium* (PREP).

Samples were analyzed adopting a multi analytical petrochemical and mineralogical characterization procedure, to describe the raw materials and reaction products constituting the mortars. In detail, the materials were analyzed by Polarized Light Microscopy on thin sections (PLM), Scanning Electron Microscopy with Energy Dispersive Spectroscopy (SEM–EDS) and Quantitative X-Ray Powder Diffraction (QPA-XRPD) of binder-concentrated samples.

Finally, the provenance of the pozzolanic pyroclastic clasts observed in two samples was determined by coupling QPA-XRPD and SEM–EDS point-analyses with X-Ray Fluorescence (XRF) and Laser-Ablation Inductively-Coupled-Plasma Mass-Spectrometry (LA-ICP-MS).

## Results

### Mortar types and reaction products

**Figure 2:**
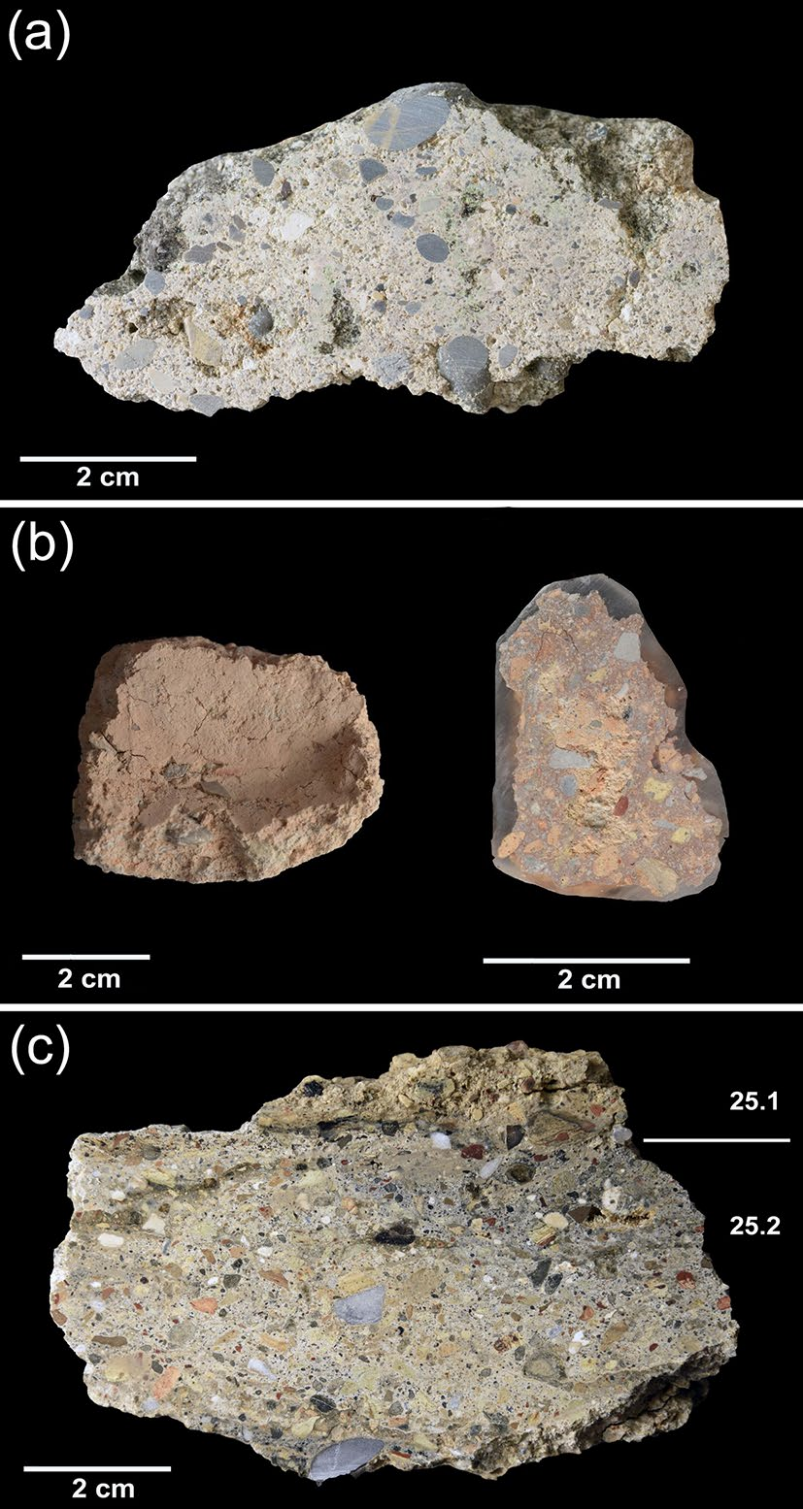
Cross sections of representative samples of the three groups of mortars. (**a**) WM_9 (Group 1); (**b**) PREF_13B (Group 2); (**c**) PREP_25 (Group 3).

Three groups of samples with similar features (Fig. 2a,b,c) were identified and described by PLM (Table. 1).

**Table 1:** Composition and binder to aggregate proportions defined by Polarized Optical Microscopy analyses on thin sections of the samples collected from the theatre. Legend: ●●●●●: extremely abundant (> 50% area); ●●●●: very abundant (~ 30–50% area); ●●●: abundant (~ 16–29% area); ●●: frequent (~ 6–15% area); ●: sporadic (~ 1–5% area); tr: traces (< 1% area); L: Lime; C: Clay; –: below detection limit.

Group	Sample	Binder	Aggregate																			
		Lime lumps and unburned relicts	Abundance	Composition	Average coarse fr. (mm)	Average fine fraction (mm)	Carbonate sand (limestones and dolostones)	Chert	Quartz	Limestone chips	Sandstone	Clay	Feldspars	Fired-clay fragments	Fired-clay powder	Pyroclastic rocks	Recycled mortars	Mica	Bioclasts	Organic fibers	Porosity	Binder:Aggregate
1	PREF_1	●	●●	L	5.08	0.33	●●●●●	●●	●●	●●	●	●	–	–	–	–	–	tr	–	–	●●	1:2.5
	PREF_4	●●	●●●	L	3.80	0.34	●●●●	●●	●	–	●	●	tr	–	–	–	–	tr	–	–	●●	1:1.5
	PREF_7	●●	●●●	L	3.24	0.38	●●●	●●●	●	–	●	●	–	tr	–	–	–	tr	–	–	●	1:1.5
	PREF_8	●	●●●	L	3.35	0.34	●●●	●	●●	●	●	●	tr	–	–	–	–	tr	–	–	●	1:1.5
	PREF_9	tr	●●●	L	4.20	0.30	●●●●	●●	●	●	tr	tr	–	–	–	–	–	–	–	–	●	1:2
	PREF_10	●	●●●	L	3.43	0.35	●●●●	●●	●	●	–	●	–	–	–	–	tr	–	–	–	●●	1:1.5
	PREF_11	–	●●●	L	3.11	0.32	●●●●	●	●	–	●	tr	–	–	–	–	●	tr	–	–	●	1:1.5
	PREF_12	●	●●●	L	3.40	0.35	●●●●	●●	●	–	tr	tr	–	–	–	–	–	–	–	–	●●	1:1.5
	WM_1	●	●●●	L	4.12	0.40	●●●●	●●	●	–	–	●	–	–	–	–	–	–	tr	–	●●	1:1.5
	WM_3	●	●●●	L	3.56	0.42	●●●●	●●	●	–	●	●	–	–	–	–	–	–	tr	–	●●	1:1.5
	WM_6	●	●●●	L	3.98	0.39	●●●●	●	●	–	tr	tr	tr	–	–	–	–	tr	–	–	●	1:1.5
	WM_7	●●	●●●	L	3.51	0.38	●●●	●●	●	–	–	–	–	–	–	–	–	–	–	–	●●	1:2
	WM_8	●	●●	L	6.35	0.37	●●●●●	●	●	–	–	tr	–	–	–	–	–	–	–	–	●●●	1:2.5
	WM_9	●	●●	L	5.34	0.49	●●●●	●●●	●	–	tr	–	–	tr	–	–	–	–	tr	–	●●	1:2.5
	WM_10	●	●●●	L	3.98	0.41	●●●	●●	●	●●	●	–	tr	–	–	–	–	tr	–	–	●●	1:2
	WM_11	●	●●●	L	3.55	0.43	●●●	●●	●	–	●	–	tr	–	–	–	–	–	–	–	●●	1:1.5
	WM_12	●	●●	L	6.63	0.39	●●●●●	●●	tr	–	●	●	tr	–	–	–	–	–	–	–	●	1:2.5
	WM_13	tr	●●●	L	4.10	0.42	●●●●	●●	●	–	tr	–	–	–	–	–	–	–	–	–	●●	1:1.5
	WM_14	●●	●●●	L	3.23	0.44	●●●	●●	●	–	●●	–	tr	–	–	–	–	–	tr	–	●●	1:2
	WM_15	●	●●●	L	4.10	0.46	●●●	●●●	●	–	●	–	–	–	–		–	–	tr	tr	●●	1:2
	WM_35	–	●●●	L	3.40	0.38	●●●	●	●●	●	–	tr	tr	●	●	–	–	tr	tr	–	●	1:1.5
2	PREF_13A	●	●●●	L	2.20	0.27	●●●	●	●	–	tr	●	tr	●●	●	–	●	tr	–	–	●	1:1.5
	PREF_13B	tr	●●●	L	2.36	0.24	●●	●	tr	–	tr	●	–	●●●	●●	–	●	–	–	–	●	1:1.5
	PREF_14	●●	●●●	L/C	3.37	0.38	●●	●	●	–	–	●●●	–	●●	●	–	tr	tr	–	–	●	1:2
3	PREP_25.1	–	●●●	L	3.11	0.34	●	tr	●	tr	–	–	tr	●●●	●	●●	–	–	–	–	●	1:2
	PREP_25.2	●	●●●	L	6.54	0.36	●	tr	●	●	–	tr	tr	●●	●	●●●	–	tr	–	–	●	1:1.5
	PREP_53	●	●●	L	11.20	0.43	●●	●	●	●	●	●	tr	●●	●	●●	●	–	tr	tr	●	1:2

**Figure 3:**
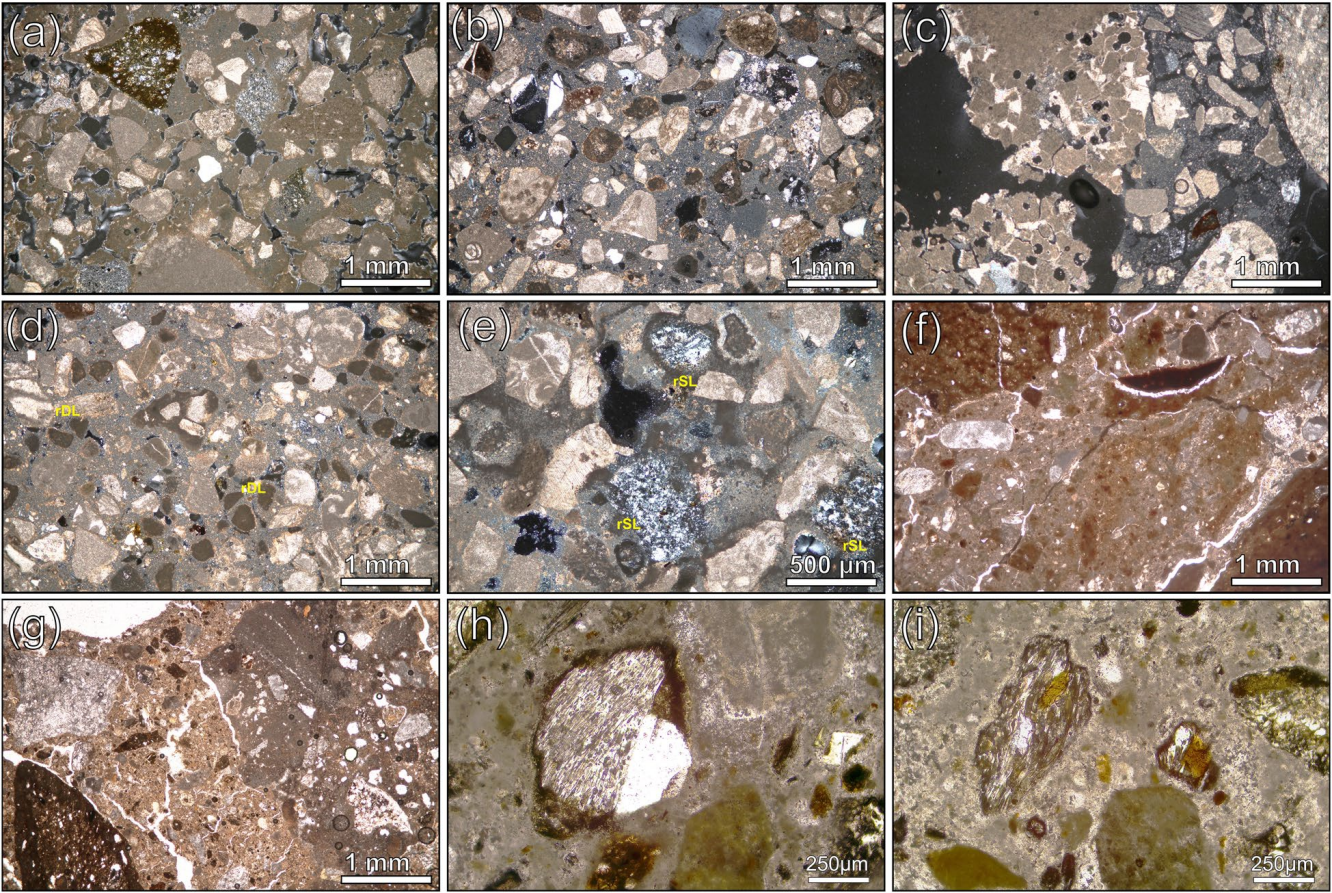
Detailed micrographs of representative samples by Polarized Light Microscopy (PLM), both in crossed (XN) and parallel (PN) nicols. (**a**) WM_11 (XN). The lime matrix displays high birefringence colors, indicating a complete carbonation of the binder. The aggregate is mainly represented by medium-to fine clasts of limestones or dolostones and silicate sand, with chert prevailing over quartz; (**b**) PREF_11 (XN). The lime matrix displays low birefringence colors, indicating an incomplete carbonation of the binder; (**c**) WM_15 (XN). Unburned fragment of dolomitic limestone, with relicts of rhombohedral crystals of dolomite. The lime matrix displays low birefringence colors; (**d**) WM_12 (XN), reacted clast of dolostones (rDL) having low birefringence due to dedolomitization phenomena; (**e**) PREF_8. Reaction rims around clasts of chert (rSL), having low birefringence; (**f**) PREF_13B (PN). The *terracotta* component of the sample is abundant, with scattered coarse clasts (at the corners of the picture) and diffused *terracotta* powder, intimately mixed with the lime matrix; (**g**) PREP_13A (PN). On the right, a coarse fragment of recycled mortar; (**h**) PREP_25 (PN). A micrometric clast of pumice, with a K-feldspar (sanidine) phenocryst. The reacted rim is detectable by the low birefringence; (i) PREP_25 (PN). Micrometric clasts of pumice, with biotite phenocrysts.

Group 1 reunites most of the samples that can be macroscopically described as lime-based mortars rich in gravel. The binder matrices are carbonate-based, micritic and generally homogeneous (Fig. 3a), sometimes displaying areas with low birefringence (Fig. 3b). Lime lumps are scarce but unburned relicts of limestones and dolostones (Fig. 3c)^47^ demonstrate that lime was obtained by calcination of these lithotypes, which constitute the core sedimentary outcrops of the Friuli Venezia Giulia region^48–50^. The porosity is moderate and constituted by prevalent vughs/vesicles voids.

The coarse fraction of the aggregate consists primarily of subrounded grains having a GSD ranging in the field of the fine gravels (6.6 to 3.1 mm, with SD = 0.9)^51^. The lithology of the clasts includes dolostones, bioclastic micritic limestones and crystalline limestones. Angular fragments of chert are also frequent, while sandstone gravels are occasional. Scattered *terracotta* fragments are present in sample WM_35. The fine fraction of the aggregate consists of subrounded grains having a GSD ranging in the field of medium to fine sands (0.49 to 0.30 mm, with SD = 0.05)^51^, constituted by clasts of limestone and dolostone, a subordinate fraction of chert and a scarce fraction of quartz and quartzites. Micas, phyllosilicates and feldspars are present in very low amounts.

The aggregates used in these mortars have a local provenance, as their petro-mineralogical nature corresponds with the sediments of the Isonzo-Natisone-Torre fluvial networks^52^, regularly employed as raw aggregate in mortars^53–56^ and wall-paintings^57,58^ of Roman Aquileia.

**Figure 4:**
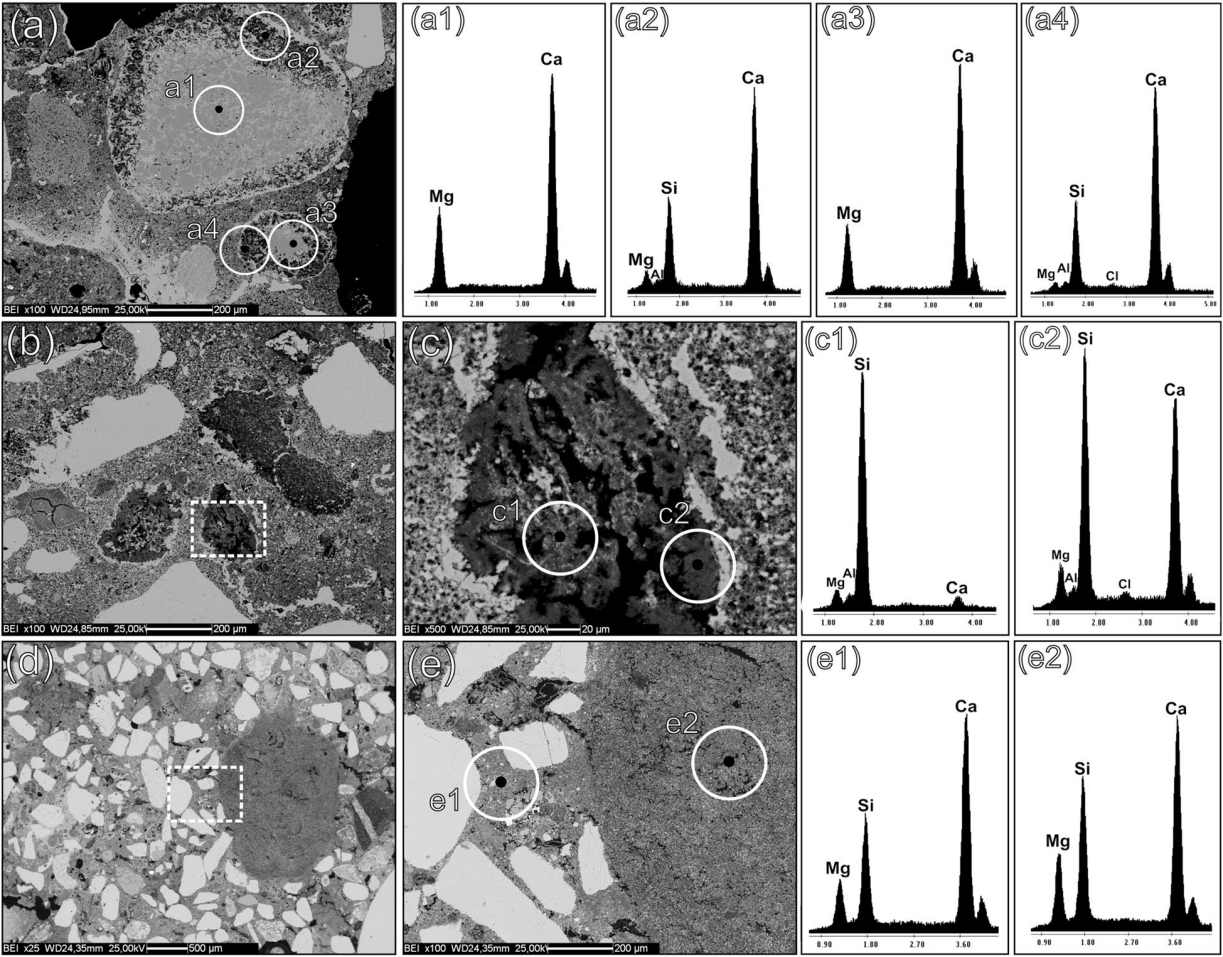
SEM–EDS analyses on representative samples of Group 1, showing reacted dolostones and chert clasts displaying the development of M-S–H gels. Backscattered electrons (BSE) acquisitions. (**a**) WM_3, altered clasts of dolostone; (**a1**) EDS spectrum of the unreacted core of a dolostone clast; (**a2**) EDS spectrum of the Mg-depleted rim of the clasts, with local enrichment in M–(A)–S–H; (**a3**) EDS spectrum of the unreacted core of another dolostone clast; (**a4**) EDS spectrum of the Mg-depleted rim of the clasts, with local enrichment in M–(A)–S–H; (**b**) WM_3, altered clasts of chert; (**c**) magnification of the dashed area at fig. (**b**); (**c1**) EDS spectrum of a feebly altered core of a clast of chert; (**c2**) EDS spectrum of a reacted area of the clast, indicating a local development of M–(A)–S–H and a likely occurrence of C–S–H through reaction with the lime component; (**d**) PREF_12, lime matrix of the sample with a lime lump on the right; (**e**) magnification of the dashed area in fig. (**d**); (**e1**) EDS spectrum of an area of the binder matrix displaying M–S–H development; (**e2**) development of M–S–H within the micropores of a lime lump.

In most of the samples of this main group, low birefringence edges around clasts of dolostones were detected, suggesting de-dolomitization phenomena^59^ (Fig. 3d). As determined by SEM–EDS analyses, the outer rims of these clasts are usually Mg-depleted and Si-enriched (Fig. 4a,a1,a2,a3,a4). Chert aggregates are also reacted (Fig. 3e), with particularly evident Ca and Mg enrichments around the edges (Fig. 4b,c,c1,c2).

In these samples, anomalous peaks of Si and Mg, having an approximate Si:Mg proportion of 2:1, were documented by the analysis of the binder matrix and in matrix-filled micropores of the lumps (Fig. 4d,e,e1,e2). The alteration of chert and dolostones likely induced the mobilization of magnesium and silica ions that, in aqueous solution, developed into magnesium-silicate hydrates (M–S–H)^60–63^, or M–(A)–S–H when free aluminum was available^62^. Precipitation of M–S–H phases usually occurs in alkaline environments, fostering alkali-silica reaction (ASR) and alkali-carbonate reaction (ACR)^64,65^. The use of brackish water in mortars production may have further favored the process kinetics, determining a pH rise through Na^+^ and SO_4_^2−^ enrichments^62,66–68^, as recently attested also in ancient binding materials^69,70^.

**Figure 5:**
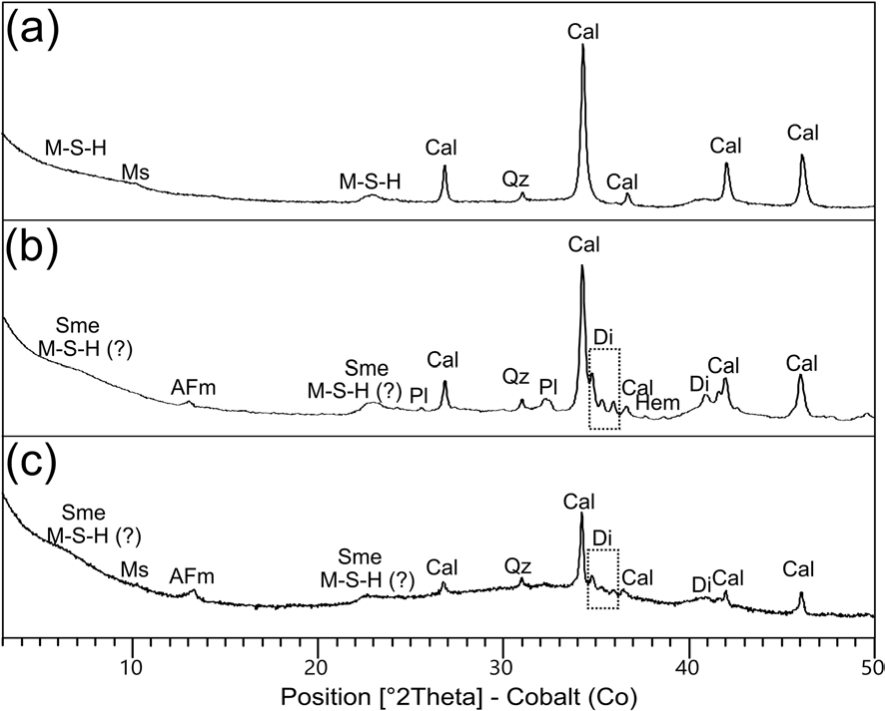
XRPD patterns of binder-concentrated fractions from representative samples of the three mortar groups, with indication of the main mineral phases (mineral abbreviation labelled according to^104^). (**a**) sample PREF_12 (Group 1); (**b**) sample PREF_13B (Group 2); (**c**) sample PREP_25, lower layer (Group 3).

In the XRPD pattern of the binder-concentrated fraction of sample PREF_12 (Fig. 5a), broad low-angle peaks ascribable to feeble-crystalline M-S–H phases of phyllosilicate structure were described through the structural pattern of a turbostratically-disordered smectite clay^60,62,63^. The high amorphous fraction (42.9 wt%) of this sample could be primarily related to a gel-like M–(A)–S–H/M–S–H phase (Supplementary Table 1). The remaining phases are calcite, to be primarily related with the carbonated binder (with a possible feeble concentration of liquid-suspended fine-grained limestone aggregates), quartz and muscovite, to be related with finely liquid-suspended particles from the aggregate fraction.

Group 2 includes three samples (PREF_13A, 13B and 14), collected from the lower part of the *opus caementicium* foundation of the *ima cavea*, that can be described as *terracotta*-rich mortars. The binder of PREF_13A and 13B is calcic, while it is made of a mix of lime and clay in PREF_14, with proportions around 1:1. Lime lumps are abundant, especially in PREF_14. The porosity in these samples is very low and constituted by scattered planar-type voids. The coarse fraction of the aggregate is exclusively represented by angular millimetric *terracotta* fragments (from 2.2 to 3.4 mm, with SD = 0.5), while the *terracotta* dust (< 75 µm) is abundant only in PREF_13B (Fig. 3f) and intimately mixed with lime (*cocciopesto*). Recycled fragments of mortars are also present in PREF_13A and PREF_13B (Fig. 3g). A subordinate fraction of the aggregate is composed of medium to fine local carbonate sand, with subordinated grains of chert and quartz/quartzites. Cherts’ and dolostones’ alteration phenomena were detected by PLM also in these samples.

The extent of hydraulic reaction of the fine *terracotta* rich mortar PREF_13B was determined by the XRPD analysis of the binder-concentrated sample (Fig. 5b), that showed a slight development of AFm phase (2.4 wt%), a hydrated calcic ferro-aluminate of the C–A–H type^71^. In the XRPD pattern, calcite primarily refers to the carbonated lime, while diopside, plagioclase and hematite are fine crystalline liquid-suspended compounds of the *terracotta* aggregates. Also quartz, barely detectable, is attributable to intrusions from fine liquid-suspended quartz/chert aggregates. As for sample PREF_12, low-angle peaks described with smectitic structures are not univocally relatable to feebly crystalline M–S–H phases, as they could be related to a liquid-suspended non-dehydroxylated fraction of the *terracotta* component.

Group 3 includes the last two samples, PREP_25 and PREP_53, that display a slightly different composition. The main feature of these compounds is the presence of pyroclastic grains (mainly pumices), constituting a relevant aggregate component in sample PREP_25, while they are scattered in PREP_53.

PREP_25 is split into two layers having similar composition. The upper one (25.1) has a carbonate-based micritic binder. The porosity is very low and represented by scattered planar-type voids. The aggregate fraction is moderately sorted (coarse fraction = 3.11, fine fraction = 0.34, SD = 1.4) and mainly composed of millimeter-sized *terracotta* fragments, while pyroclastic aggregates represent a subordinate component. Approximately 1/3 of the aggregate fraction consists of sands compatible with the lithology of local fluvial sediments.

In the lower layer (25.2), the sorting between the coarse and the fine fraction of the aggregate is lower (from 6.5 to 0.34 mm, with SD = 3.1) and the pyroclastic component prevails over the *terracotta* fraction.

Sample PREP_53 is coarser in composition than the previous one and the sorting of the aggregate is very low (coarse fraction = 11.2, fine fraction = 0.43, SD = 5.4). The sample displays an array of pluri-millimetric and centimetric aggregates, including coarse gravels, angular stone chips, coarse *terracotta* fragments and various organic elements (i.e. straw and walnut shells). In this sample, the volcanic fraction is larger in size (sometimes around 0.5 up to 1.0 mm).

**Figure 6:**
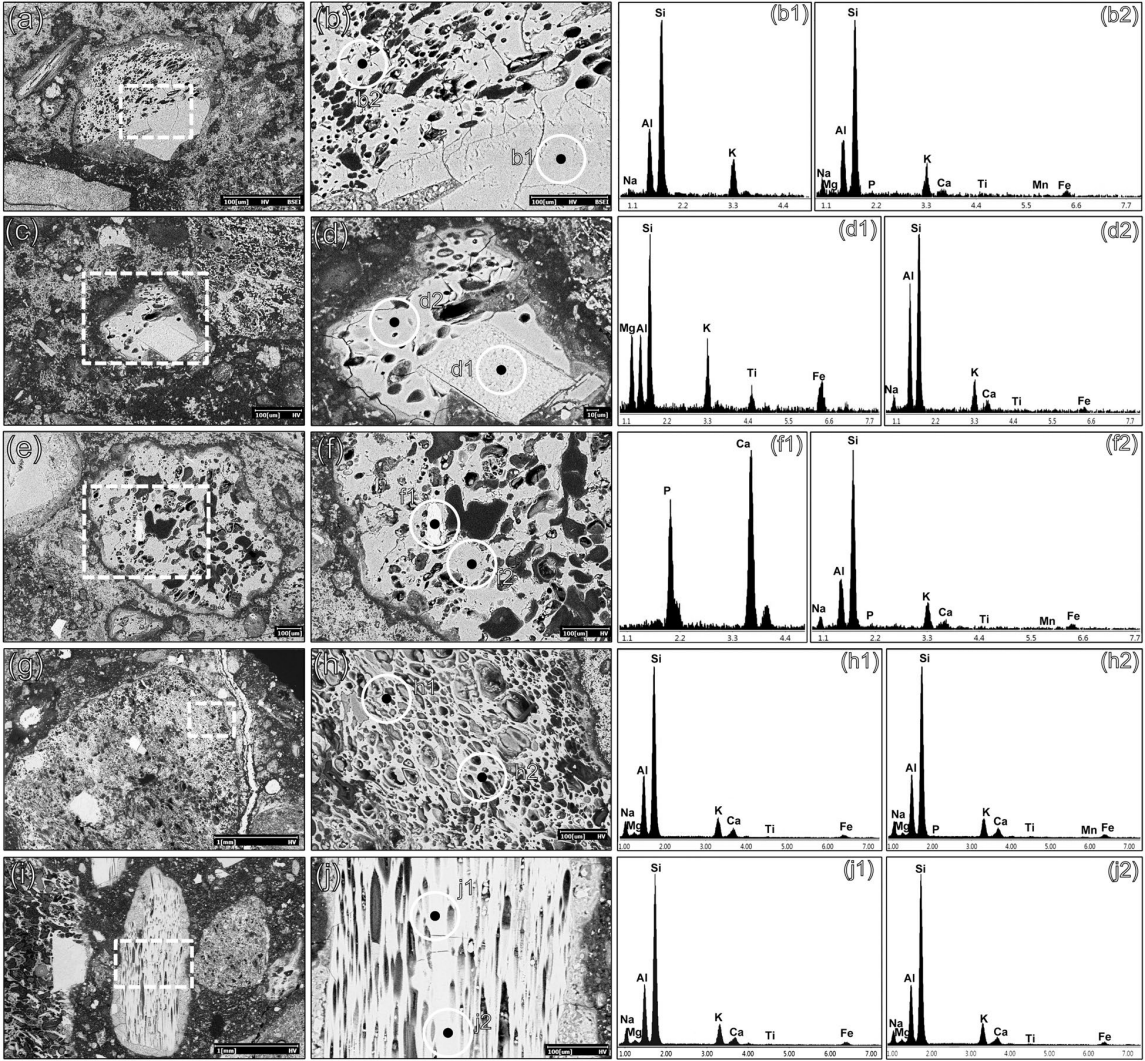
SEM–EDS analyses on representative samples of pumice in sample PREP_25. Backscattered electrons (BSE) acquisitions. (**a**) pumice clast *c4*; (**b**) magnification of the dashed area in fig. (**a**); (**b1**) EDS spectrum of a K-feldspar phenocryst (sanidine); (**b2**) EDS spectrum of unaltered volcanic glass; (**c**) pumice clast *c5*; (**d**) magnification of the dashed area in fig. (**c**); (**d1**) EDS spectrum of a Ti–rich biotite phenocryst; (**d2**) EDS spectrum of unaltered volcanic glass; (**e**) pumice clast *c6*; (**f**) magnification of the dashed area in fig. (**e**); (**f1**) EDS spectrum of an apatite phenocryst; (**f2**) EDS spectrum of unaltered volcanic glass; (**g**) pumice clast *g*; (**h**) High-resolution magnification of the dashed area in fig. (**g**); (**h1**, **h2**) EDS spectra of aphyric volcanic glass; (**i**) pumice clast *a*; (**j**) High-resolution magnification of the dashed area in fig. (**i**); (**j1**, **j2**) EDS spectra of aphyric volcanic glass.

In both samples PREP_25 and PREP_53, pyroclastic clasts are mainly constituted of finely ground (GSD ranges from < 75 µm to < 1.5 mm) highly vesicular glassy pumices (Fig. 3h,i). Rare phenocrysts consist of scattered sanidine (Fig. 6, a,b,b1), biotite (Fig. 6, c,d,d1) and apatite (Fig. 6e,f,f1). The texture of the pyroclastic grains is aphyric (Fig. 6g,h,h1,h2,i, j,j1,j2). PREP_53 presents a single clast of tuff (clast *w*), recognized by QPA-XRPD for its characteristic mineralogical composition (see the next paragraph).

The binder of sample PREP_25 (lower layer) exhibits an inhomogeneous texture around the pozzolanic clasts (both pumices and *terracotta* fragments), with low birefringence areas where the local development of C–A–S–H/C–A–H likely occurred. Indeed, the XRPD pattern of the binder-concentrated fraction of sample PREP_25 reveals the development of crystalline AFm phase (3.8 wt%), while most part of C–A–H/C–A–S–H has a gel-like structure, as suggested by the high amorphous rate (74.1 wt%). The low amount of calcite (~ 15 wt%) indicates that just a limited part of the lime component underwent complete carbonation (Fig. 5c).

The pyroclastic aggregates observed in the preparation mortars of the *orchestra* and *hyposcaenium* were surely imported in Aquileia, as no volcanic units characterized by explosive activity outcrop in the Friuli Venezia Giulia region^49,50^ (see also Supplementary Figure 3).

### Provenance of pyroclastic rocks

Detailed mineralogical, petrographic and geochemical analyses of the pyroclastic clasts observed in samples PREP_25 and PREP_53 were crucial for determining their provenance. A sub-centimetric clast of tuff from sample PREP_53 (clast *w*) was mechanically separated from the mortar and analyzed by QPA-XRPD and XRF. The millimetric to sub-millimetric clasts of pumice in samples PREP_25 and 53 were investigated by SEM–EDS and LA-ICP-MS point-analyses on polished ~ 1 mm thick sections (for instrumental and acquisition parameters see "Methods").

**Figure 7:**
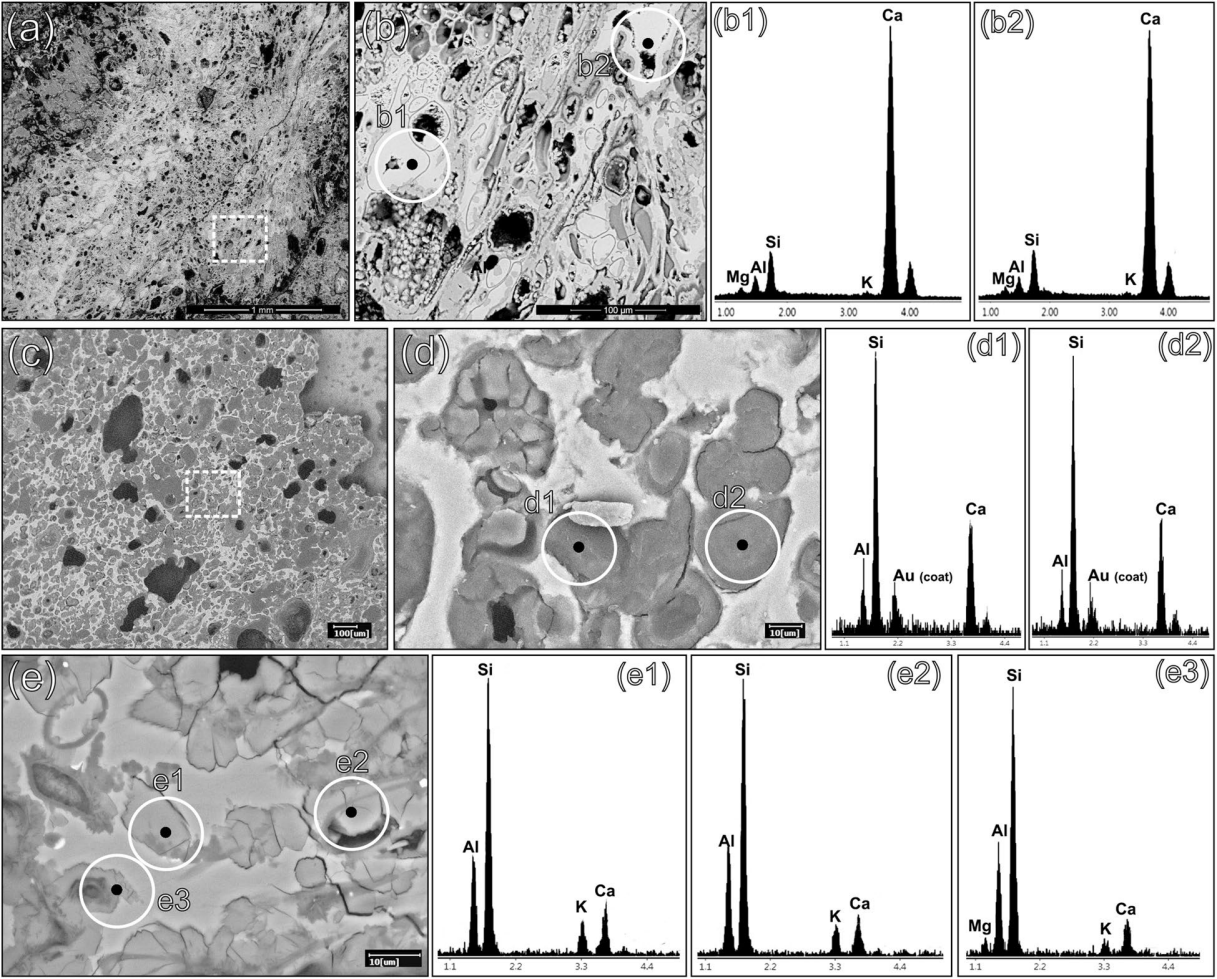
SEM–EDS analyses of reacted pumices in samples PREP_25 and PREP_53. Backscattered electrons (BSE) acquisitions. (**a**) A reacted clast of pumice in sample PREP_25; (**b**) magnification of the dashed area in fig. (**a**); (**b1**, **b2**) EDS spectra of a reacted area of the volcanic glass, with vesicles filled of calcic compounds, likely related to calcium carbonates (calcite or vaterite) from the binder; (**c**) A reacted area of a clast of pumice in sample PREP_53; (**d**) magnification of the dashed area in fig. (**c**); (**d1**, **d2**) EDS spectrum of a C–A–S–H enriched zone, developed from the leached volcanic glass and filling the vesicles; (**e**) zeolitized volcanic glass in a clast of pumice in sample PREP_25; (**e1**, **e2, e3**) EDS spectra likely referred to anthropogenic phillipsite formed by pozzolanic reaction.

**Figure 8:**
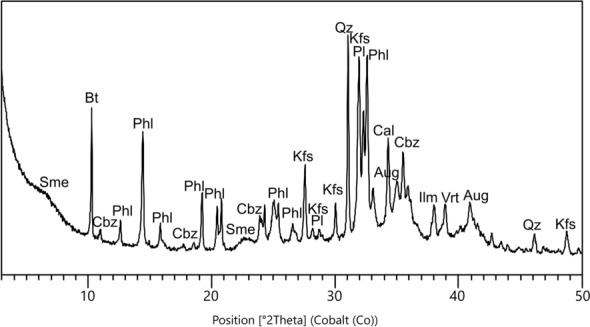
XRPD spectrum of the sub-centimetric clast of tuff (w) in sample PREP_53, with indication of the main mineral phases (mineral abbreviations labelled according to^104^).

A pre-screening of major chemical elements of the pyroclastic aggregates was performed by multiple semiquantitative SEM–EDS point-analyses. However, the pervasive pozzolanic reaction determined a relevant alteration of the original geochemical fingerprint in most of the rocks. Indeed, both outer rims and inner vesicles of the volcanic clasts are generally Ca-enriched (Fig. 7a,b,b1,b2 or filled with C–A–S–H (Fig. 7c,d,d1,d2). Alteration was detected also by the XRPD analysis of tuff clast *w* (Fig. 8, Supplemenatry Table 2), where enrichments in calcite, related to carbonated lime infills, and vaterite, representing a metastable anthropogenic product formed by decalcification and re-carbonation of CaCO_3_ during pozzolanic reaction^72–75^, were detected. The presence of quartz might be accidental as it probably represents a silicate aggregate from the mortar (i.e. chert).

All the remaining phases can be attributed to the original mineralogy of the volcanic grain. Phillipsite (11.1 wt%) and chabazite (3.3 wt%) are common authigenic zeolites nucleating through hydrothermal processes in the ultrapotassic products of the Roman Comagmatic Region and they are recurrently detected in the zeolitized tuffs of the Phlegraean Fields^12,76–78^. However, the in situ growth of anthropogenic phillipsite from leached volcanic glass was observed by SEM–EDS in several pumice samples as a consequence of pozzolanic reaction processes^29,75^ (Fig. 7e,e1,e2,e3), making the mineralogical data non-conclusive for provenance determination.

Therefore, a distinction between almost-completely reacted clasts and those preserving unaltered cores, as measured at Fig. 6(b2,d2,f2,h1,h2 and j1,j2) was necessary for further in-depth investigations.

SEM–EDS analyses, measured on unaltered cores of the aphyric volcanic glass were obtained for pumice clasts in samples PREP_25 (clasts *c1, c2, c3, c4, c5, c6, c7, a, e, f, g, l, o, q, r,*) and PREP_53 (clast* z*).

All clasts present a coherent geochemical profile of major elements (Supplementary Table 3), with average values of Na_2_O = 5.8 wt% (SD = 0.75), MgO = 1.08 wt% (SD = 0.46); Al_2_O_3_ = 19.0 wt% (SD = 0.41), Cl_2_O = 0.82 wt% (SD = 0.17), K_2_O = 7.42 wt% (SD = 0.79), CaO = 3.43 wt% (SD = 0.99); TiO_2_ = 0.78 wt%. (SD = 0.42). Only the average values of SiO_2_ = 57.8 wt%, with SD = 2.34, and Fe_2_O_3_ = 3.31 wt%, with SD = 1.17, are characterized by an higher degree of variability.

The resulting profiles of major elements were compared with the geochemical fingerprint of the volcanic products of the Plio-Quaternary magmatic activity of the Italian peninsula and islands, as reported in the scientific literature. The TAS (Total Alkali *vs.* Silica) diagram^79^, reporting the relation between alkaline elements (Na_2_O + K_2_O) and silica (SiO_2_), offered a first discrimination of the geochemical distribution of volcanic clasts. This tool is frequently adopted in the study of volcanic aggregates in archaeological mortar-based materials analyzed by SEM–EDS^10,17,33,34,36,37^.

**Figure 9:**
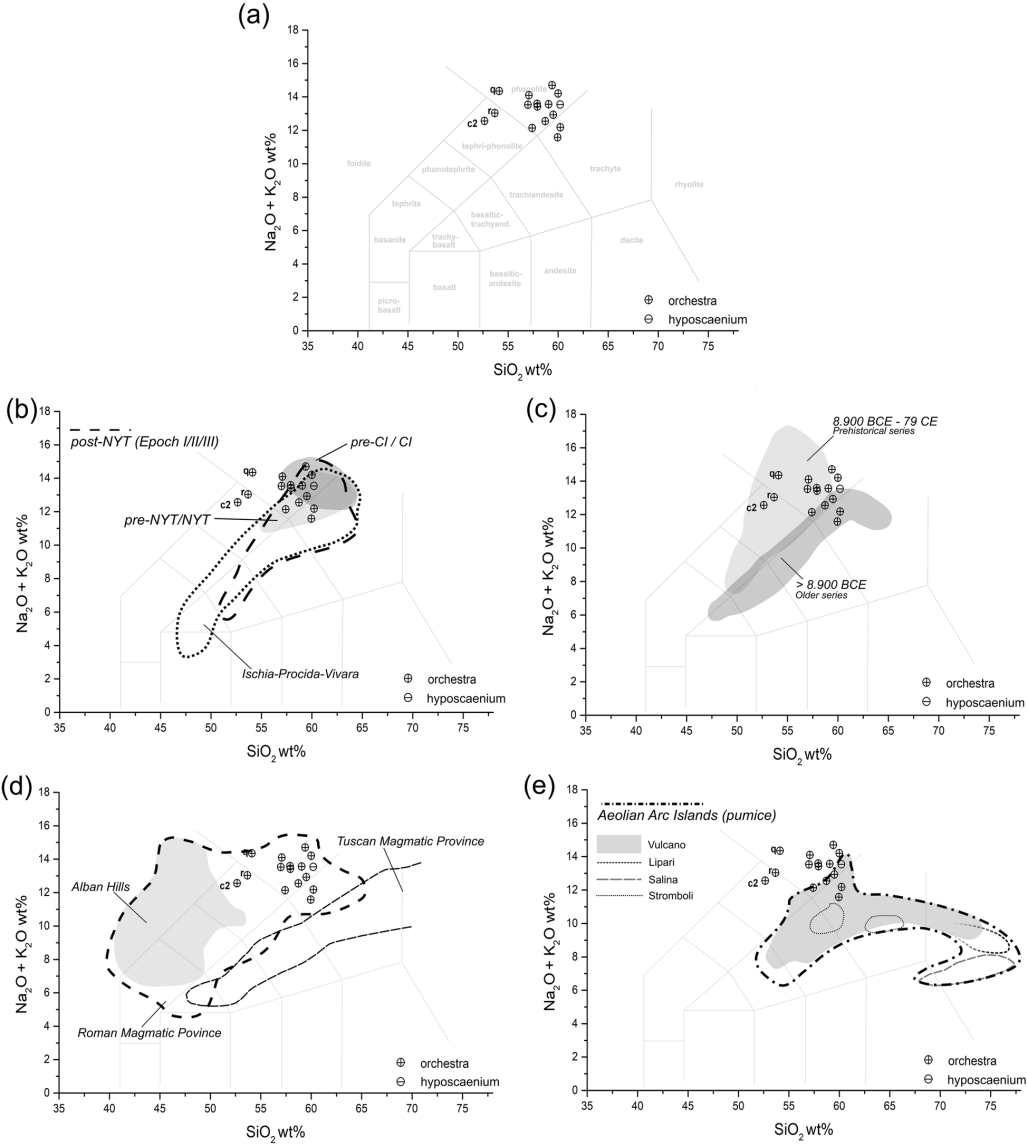
TAS (Total Alkali vs Silica) scatterplots of pumices (volcanic glass) in samples PREP_25 and PREP_53. (**a**) Samples’ distribution according to volcanic rocks’ chemistry (after^79^); (**b**) samples’ distribution according to rock chemistry of the Phlegraean volcanic products regarding the main eruptive events of the pre- and Campanian Ignimbrite (pre-CI/CI), pre- and Neapolitan Yellow Tuff (pre-NYT/NYT), post-NYT (Epoch I, II, II, according to^82^), and Phlegraean-correlated volcanoes of Ischia and Procida-Vivara (compositional fields edited from^11,12,80,81^); (**c**) samples’ distribution in relation to the three main eruptive facies of the Somma-Vesuvius volcanic activities (compositional fields edited from^11,12,81^); (**d**) samples’ distribution in relation to the fields occupied by the products of the Roman and Tuscan Magmatic provinces (compositional fields edited from^11,83,84^); (**e**) samples’ distribution in relation to the fields occupied by the pyroclastic products of the Aeolian Arc Isles (compositional fields based on raw data from^85^).

The clasts in samples PREP_25 and 53 primarily present a phonolitic composition (Fig. 9a), and subordinately trachytic (clasts *g, o*) and tephri-phonolitic (clasts *r, c2*). This profile is compatible with most of the volcanic products of the Campanian magmatic province, including the alkaline and highly-alkaline series of the main Phlegraean eruptions (pyroclastic products)^81^ comprising the pre- and Campanian Ignimbrite (pre-CI/CI), pre- and Neapolitan Yellow Tuff (pre-NYT/NYT), post-NYT (Epoch I, II, II, according to 82] as well as the Phlegraean-correlated volcanoes of the islands of Ischia and Procida-Vivara (pyroclastic products) (Fig. 9b). The analyzed clasts marginally overlap with the older and prehistorical series of Somma-Vesuvius’ tephra (Fig. 9c)^81^. Only clasts *q, r, c2* in sample PREP_25 by the TAS do not overlap with the area of the Phlegraean Fields and Ischia/Procida-Vivara, but they entirely fall within the field of the highly alkaline pumices and ashes of the Somma-Vesuvius prehistorical series pre-dating 79 CE. The main difference of these clasts (Supplementary Figure 4) from the main core is the lower concentration of SiO_2_ (< 55.0 wt%) and the feebly higher concentration of MgO (> 1.0 wt%) and K_2_O (> 8.0 wt%).

All analyzed clasts are geochemically incompatible with the highly alkaline cinerites of the Colli Albani (*harenae fossiciae*) and with most of the other products of the Roman and Tuscan magmatic provinces^11,83,84^ (Fig. 9d). Correspondences in the TAS diagram can be observed with the pyroclastic products of the Aeolian Arc Isles, in particular with certain pumices of Vulcano^85^ displaying a phonolitic chemism (Fig. 9, e).

**Figure 10:**
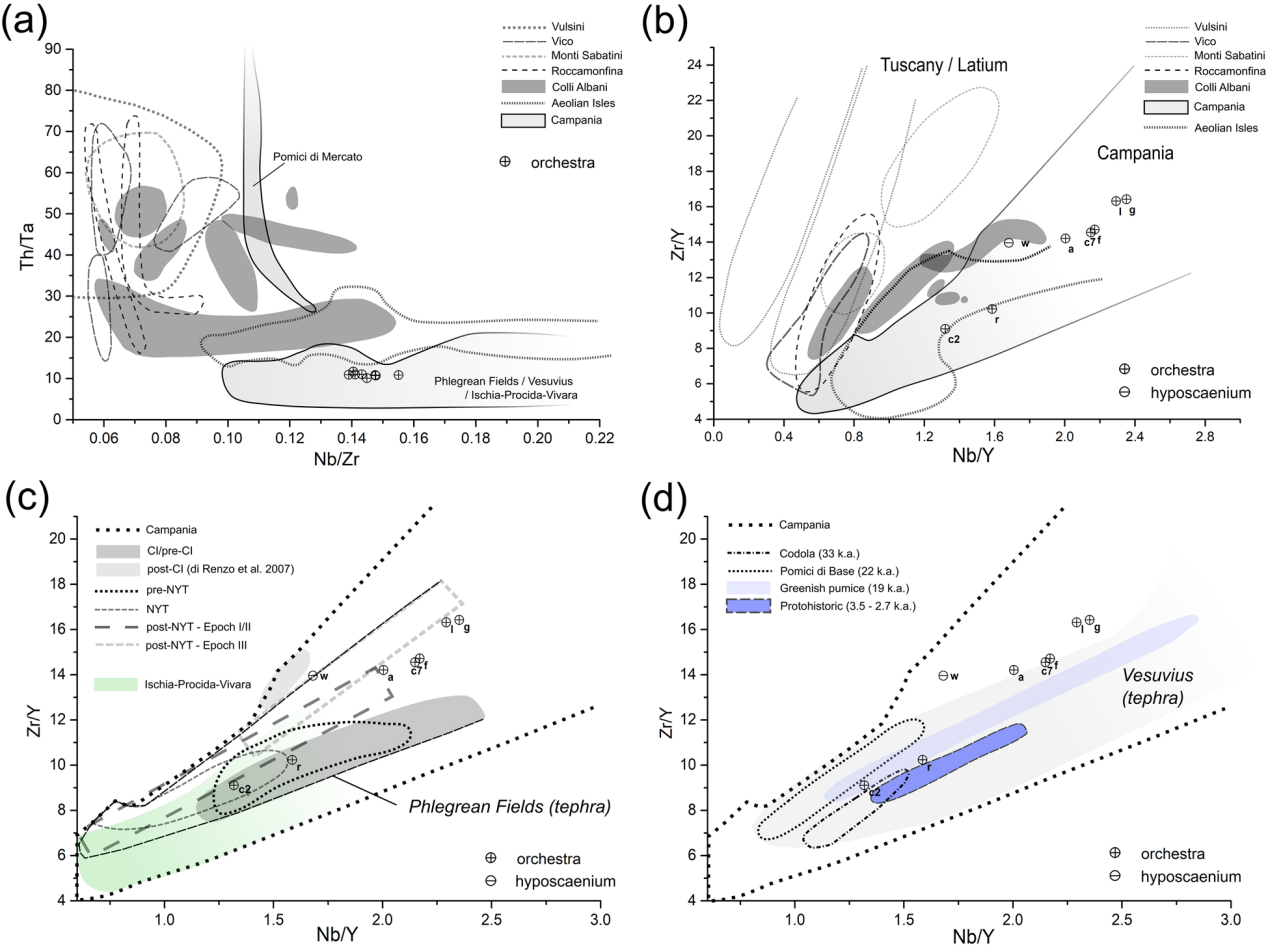
Trace elements’ scatterplots of the volcanic grains in samples PREP_25 (pumices) and PREP_53 (tuff); (**a**) Nb/Zr vs Th/Ta scatterplot of clasts’ samples in relation to the fields occupied by the Roman, Tuscan and Campanian magmatic provinces (compositional fields edited from^7,8,10,22,80,89,90^), and Aeolian Arc Islands’ volcanic products (compositional fields based on raw data from^85–87^); (**b**) Nb/Y vs Zr/Y scatterplot of clasts’ samples in relation to the fields occupied by the Roman, Tuscan and Campanian magmatic provinces (compositional fields edited from^80,89,90^), and the Aeolian Arc Island's products (compositional fields based on raw data from^85–87^); (**c**) Nb/Y vs Zr/Y scatterplot of clasts’ samples in relation to the fields occupied by volcanic products of the Phlegraean Fields main eruptions (according to^82^; compositional fields edited from^80,89,90^), and Phlegraean-correlated products (pumices and scorias) of Ischia/Procida-Vivara (compositional fields based on raw data from^91,92^); (**d**) Nb/Y vs Zr/Y scatterplot of clasts’ samples in relation to the fields occupied by volcanic products of Somma-Vesuvius main pre-79CE eruptions (according to^93^, compositional fields edited from^80^).

Considering the high variability of the TAS, trace elements were crucial to confirm the exact provenance of the volcanic pozzolans; traces were acquired by LA-ICP-MS analyses of the pumice clasts *c2, c7*, *a, f, g, l, r,* in sample PREP_25 (Supplementary Table 4 and Supplementary Figure 5) and XRF analysis of the tuff clast *w* in sample PREP_53 (Supplementary Table 5). Considering REE and HFSE, the ratios among Zr, Y, Nb, Th, and Ta are usually adopted in scientific literature for determining the geochemical fingerprint of the Italian Plio-Quaternary magmatism^7,8,10,22,81,89–91,95^. In the analyzed clasts, most of these trace elements are characterized by a certain variability, with Zr = 513 ppm (SD = 277), Y = 37 ppm (SD = 17), Nb = 75 ppm (SD = 39), Th = 48 ppm (SD = 27) and Ta = 4 (SD = 2). However, on the basis of the analysis of Zr/Y versus Nb/Y and Nb/Zr versus Th/Ta (with the exception of clast *w*, as Ta was not acquired by XRF), all clasts systematically plot in the field of the Campanian magmatic province (Fig. 10a,b), with weak overlaps with the fingerprint of the volcanic products of other magmatic districts potentially corresponding with the frame here considered . For some of the analyzed clasts (*r*, *c2*), a certain overlap can be detected on the Zr/Y vs Nb/Y scatterplot with the tephra fingerprint of the Aeolian volcanoes^85–87^, but this is not observed in Nb/Zr vs Th/Ta diagram, making the Aeolian Isles incompatible for the provenance.

In detail, clasts *a, f, l, g, w *and *c7* completely overlap with the area of the Phlegraean Fields, with a close correspondence with the pyroclastic products of the post-NYT formations (Epoch III, according to^82^); *c2* and *r* clasts overlay the fields of the pyroclastic products of the older Phlegraean eruptions (pre-CI and CI, pre-NYT and NYT) as well as with the vulcanism of Ischia and Procida-Vivara (Fig. 10c). Moreover, on the basis of the Zr/Y vs Nb/Y scatterplot, strong matches with the older Vesuvian eruptions (Codola, Pomici di Base, Greenish pumice, according to^93^) and with the Proto-historic series (3.5–2.7 k.a.^93^) can be observed (Fig. 10d). The correspondence of these two clasts with the Proto-historic series of Somma-Vesuvius is arguable also on the basis of TAS scatterplot. The remaining clasts, on the other hand, falls substantially out of the field of Somma-Vesuvius fall products older than 79 CE.

Therefore, by combining mineralogical and geochemical results, the volcanic outcrops of the Bay of Naples can be proposed for the provenance of the volcanic clasts in samples PREP_25 and 53. Most of them (clasts *a, f, l, g, w* and *c7*) report strong correlations with the younger pyroclastic products of the Phlegraean eruptions (post-NYT), while, as observed through TAS profiles, the association with Somma-Vesuvius is possible for some clasts (*r, c2* and possibly* q*), even though the provenance from the Phlegraean Fields (comprising the isles of the Bay of Naples) cannot be ruled out.

## Discussion and conclusions

The analyses on the mortar samples from the theatre of Aquileia tracked one of the oldest trades and early utilizations of *pulvis puteolana* in the Roman Empire out of the Campania region. Moreover, it marks the first use of this pozzolanic powder in an overground construction far from the Bay of Naples and the first analytically proven evidence in the Roman *Cisalpina* region. Indeed, as already briefly discussed in^55^, previous studies^95,96^ on the possible presence of this product in mortar-based materials of the region were recently reconsidered. At the moment, the absence of any other evidence is not clearly attributable either to the lack of targeted research of this topic or to the actual shortage in the supply of the volcanic pozzolan from the Bay of Naples to *Cisalpina* in ancient times. Besides, the Roman trading network was likely developed enough already in the Early Imperial Age to export pozzolanic materials from Campania toward the provinces of the Empire, but further research is needed.

Nevertheless, this research highlights that, at least since the 1st Century CE (and possibly even a couple of decades earlier), the *pulvis puteolana* was not only traded for the construction of the monumental port infrastructures of the Mediterranean, but also for on-land public buildings in the framework of the ordinary civil engineering.

However, porous rocks imported from Campania were frequently adopted in the Empire for the construction of *opus caementicium* vaults but, in these circumstances, these materials were processed as decimeter-sized elements not for being used as strengthening and waterproofing aggregate for mortars, but primarily for the intrinsic lightening properties of pumices, tuffs and porous lavas. Besides, most of the evidence comes from Middle/Late Imperial high-patronaged constructions, such as the great monuments in Rome and in the main towns in the provinces^80,91,97,98^. Among them there is also Aquileia, as coarse pumices and lavas from the Phlegraean Fields and Somma-Vesuvius were used to lightweight the *opus caementicium* vaults of the Late Antique Baths of the town, which were probably built under the patronage of the Imperial family^55^.

Regarding the specific use of pozzolanic powders in the theatre of Aquileia, the volcanic pozzolans present an extremely localized occurrence within the building. This may indicate that the circulation of the material was still limited at the time, possibly because of high transportation charges, but its distribution in Aquileia did not increase even in later centuries. Indeed, in the analysis of more than 300 mortar samples from public and private buildings of the town^54^ the occurrence of the Neapolitan pozzolans was detected only in the preparation mortars of the theatre’s *orchestra* and *hyposcaenium*, together with the aforementioned *opus caementicium* vaults of the Late Imperial baths.

Therefore, it may be relevant to focus on the structures the volcanic pozzolan was used in. The material is not present in the masonry mortars nor in the foundational *opus caementicium*. Cursory consolidation systems were used for the stabilization of the theatre’s foundations as, at the bottom of the substructure, *cocciopesto* mortars with modest waterproofing properties were employed to blandly counteract the capillary rise of groundwater. The use of the volcanic pozzolan exclusively in the preparations of pavements was probably intended to ensure a waterproofed sealing, keeping the floors dry and sound against water infiltrations. Indeed, the theatre was built in the low-lying Aquileia's deltaic plain, affected by recurrent salt wedge^45,46^. Moreover, it was close to “Canale Anfora”, an artificial Roman-time channel connected to the Marano lagoon, and a land-reclamation system described by Vitruvius in lagoon-like environments of the ancient *Cisalpina*, including Ravenna and Altinum (*De Architectura*, 1.4.11)^99^. The use of volcanic pozzolans in the preparation mortars of the floors of the theatre was probably targeted at counteracting the localized water ingressions and salt wedge, characterized by basic and reducing liquid conditions. This proves the in-depth knowledge of the ancient builders of the local geomorphology and probably the presence of brackish water, possibly used for the preparation of the mortars of the building, may have fostered, through the contribution of alkalis and sulfates, the complex precipitation kinetic of calcium-based silico/aluminate hydrates and magnesium-based silico/aluminate hydrates, influencing the final microtextural features, physical properties and longevity of the investigated mortars^73,74^.

In conclusion, the way the builders carefully employed and mixed local materials with imported volcanic pozzolans where it was strictly necessary is brilliant and it somewhat resembles the best-known utilization of *pulvis puteolana* for maritime constructions. It was probably an exceptional circumstance at the time, but the overall evidence highlights the craftsmen’s resilience in adapting and reinterpreting the traditional use of the pozzolan from the Bay of Naples to deal with the recurrent water infiltrations of Aquileia’s deltaic plain.

## Methods

### Polarized light microscopy (PLM)

All mortar samples were analyzed by means of Polarized Light Microscopy (PLM) on 30 μm thin sections under a Nikon Eclipse ME600 microscope for a preliminary petro-mineralogical characterization. Mortar analysis was carried out according to the macroscopic and microstratigraphic analytical procedures described in Standard UNI 11,176:2006 “Cultural heritage—Petrographic description of a mortar”. For each sample (or for each layer in the case of multi-layered sample PREP_25), the concentration of binder, porosity and aggregates (i.e. *terracotta* fraction, sand etc.) and the binder-to-aggregate proportions were evaluated through digital image analysis performed using Image-J software^100^.

### Quantitative phase analysis by X-ray powder diffraction (QPA-XRPD)

QPA-XRPD analyses were performed on a coarse clast of pumice in sample PREP_53, mechanically separated from the sample, and on the binder-concentrated fractions of three representative samples of the mortars groups defined by OM investigations.

The binder-concentrated material from the samples was separated in water solution following the Cryo2Sonic 2.0 separation procedure^101^, custom-modified by the addition of a chelating agent (sodium hexametaphosphate 0.5 wt%) to favor the suspension of the finer, non-carbonate phases such as clay minerals and hydrate products, prone to flocculation due to their surface charges, as described in^69^.

XRPD profiles were collected using a Bragg–Brentano θ-θ diffractometer (PANalytical X’Pert PRO, Cu Kα radiation, 40 kV and 40 mA) equipped with a real-time multiple strip (RTMS) detector (PIXcel by Panalytical). Data acquisition was performed by operating a continuous scan in the 3–85 [◦2θ] range, with a virtual step scan of 0.02 [◦2θ]. Diffraction patterns were interpreted with X’Pert HighScore Plus 3.0 software by PANalytical, qualitatively reconstructing mineral profiles of the compounds by comparison with PDF databases from the International Centre for Diffraction Data (ICDD).

Then, quantitative phase analysis (QPA) was performed using the Rietveld method^102^. Refinements were carried out with TOPAS software (version 4.1) by Bruker AXS. The quantification of both crystalline and amorphous content was obtained through the addition of 20 wt% of zincite to the powders as internal standard. The observed Bragg peaks in the powder patterns have been modelled through a pseudo-Voigt function, fitting the background with a 12 coefficients Chebyshev polynomial. For each mineral phase, lattice parameters, Lorentzian crystal sizes and scale factors have been refined. Although samples were prepared with the backloading technique to minimize preferred orientation of crystallites a priori, any residual preferred orientation effect was modelled during the refinement with the March Dollase algorithm^103^. The starting structural models for the refinements were taken from the International Crystal Structure Database (ICSD).

### Scanning electron microscopy with energy dispersive spectroscopy (SEM–EDS)

SEM–EDS analyses were performed to investigate locally the chemical composition of the binder and aggregates and the reaction zones in the samples. The analytical instrument used for this analysis was a FEI Quanta 200 microscope, equipped with an Energy Dispersive X-ray detector (EDX) EDAX Element-C2B.

Chemical profiles of pyroclastic clasts were determined by means of five up to ten micro areal analyses of unaltered portions of the aphyric glass of the volcanic pumices. Acquisition was carried out on carbon coated polished thin sections of mortar samples PREP_25 and 53.

Standarless semiquantitative analysis by Team EDAX software (based on ZAF correction and factory standardization data implemented in the software) was previously tested on two NIST certificated reference materials: SRM 2066 K411 and SRM 620 (see Supplementary Table 6). These materials are glasses with a chemical composition compatible with the volcanic grains analyzed in this study. EDS analyses investigated an area of the volcanic glass having a diameter of around 1 µm, operating at 20 kV with a working distance (WD) between 11.8 and 12.2 mm.

### X-ray fluorescence (XRF)

Bulk-rock chemical analysis for major and trace elements of one clast from sample PREP_53 was performed by XRF on glass beads prepared with calcined samples diluted with Li_2_B_4_O_7_ flux (1:10 ratio), using a WDS Panalytical Zetium sequential spectrometer, operating under vacuum conditions and equipped with a 2.4 kW Rh tube. Loss on ignition (LOI) was determined separately before the XRF analysis. The calculated major elements are Si, Ti, Al, Fe, Mn, Mg, Ca, Na, K and P (expressed as a percentage of the relative oxide). The calculated trace elements (expressed in ppm) are Sc, V, Cr, Co, Ni, Cu, Zn, Ga, Rb, Sr, Y, Zr, Nb, Ba, La, Ce, Nd, Pb, Th and U. Instrumental precision (defined by repeated analysis on the same sample) is within 0.6% relative for major elements, and within 3.0% relative for trace elements. Detection limits for Al, Mg, and Na are within 0.01%, within 0.2% for Si, and within 0.005% for Ti, Fe, Mn, Ca, K, and P. Limits for trace elements are (in ppm): Sc = 3, V = 5, Cr = 6, Co = 3, Ni = 3, Cu = 3, Zn = 3, Ga = 3, Rb = 3, Sr = 3, Y = 3, Zr = 3, Nb = 3, Ba = 10, La = 10, Ce = 10, Nd = 10, Pb = 5, Th = 3, U = 3.

The material for XRF analysis was collected by mechanically separating a portion of the sample from the mortar, scraping away the interfacial zones with the binder^7^. The clast has been analyzed as bulk material without HCl bathing, as this step could affect the concentration of major and trace elements, in particular Y^8,10,22^.

### Laser-ablation inductively-coupled-plasma mass-spectrometry (LA-ICP-MS)

Spot analyses of trace elements on the selected volcanic clasts were determined by Laser-Ablation Inductively-Coupled-Plasma Mass-Spectrometry (LA-ICP-MS), using a Thermo Fisher Scientific triple quadrupole mass spectrometer coupled with a laser ablation NewWave UP 213 at the Laboratory of the Centro Interdipartimentale Grandi Strumenti (CIGS) of the University of Modena and Reggio Emilia. Data reduction was performed with an in-house Excel script using NIST612 and ML3-B reference materials as external standards. NIST610 and NIST614 reference materials were monitored during the session as unknown. Isotope ^44^Ca was used as internal standard. Laser spot size was calibrated at 50 µm and laser beam fluency at 20 microJoule for cm^2^. Analyses were carried out on ~ 1 mm thick polished sections. Analyses were performed on portions of the clasts as unaltered as possible, to collect profiles that properly represent the original geochemical fingerprint of the rocks. For each clast, the reported profiles are based on the average of three up to five spot analyses.

## Supplementary Information

Supplementary Information.

## Data Availability

All data are available in the main text or in the supplementary information.
