# Supplementary material for: Early exploitation of Neapolitan pozzolan (pulvis puteolana) in the Roman theatre of Aquileia, Northern Italy
Source: Sci Rep. 2023 Mar 13;13:4110. doi: 10.1038/s41598-023-30692-y (PMC10011562; doi:10.1038/s41598-023-30692-y)
Supplement: Supplementary file 1 — Supplementary Information. [file 41598_2023_30692_MOESM1_ESM.pdf]

Early exploitation of Neapolitan pozzolan (*pulvis puteolana*) in the Roman theatre of Aquileia, Northern Italy

Simone Dilaria*[1,2], Michele Secco[1,2], Andrea R. Ghiotto[1], Guido Furlan[3], Tommaso Giovanardi[4], Federico Zorzi[5], Jacopo Bonetto[1]

[1] Department of Cultural Heritage (DBC), University of Padova, Piazza Capitaniato 7, 35139 Padua, Italy.

[2] Inter-Departmental Research Centre for the Study of Cement Materials and Hydraulic Binders (CIRCe), University of Padova, Via Giovanni Gradenigo 6, 35131 Padua, Italy.

[3] Centre for Urban Network Evolutions – UrbNet, Aarhus University, School of Culture and Society, Moesgård Allé 20, 8270 Højbjerg, 4230-223, Denmark

[4] Department of Chemical Science and Geology, University of Modena and Reggio Emilia, Via Università 4, 41121 Modena, Italy

[5] Analysis Center and Certification Services (CEASC), University of Padova, Via Jappelli 1/A, 35121 Padua, Italy

Supplementary Information

Supplementary Figure 1 - Results of the radiocarbon dating carried out on organic materials collected from the foundation layers of the *hyposcaenium* (Oxford Radiocarbon Accelerator Unit). Further analyses and a comprehensive study of the whole set of chronological data available are in progress.

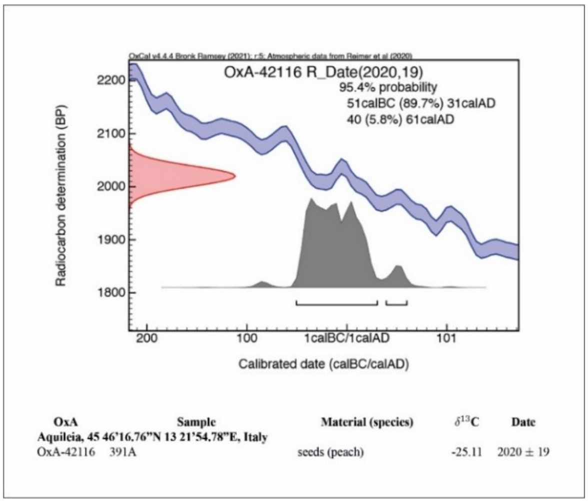

Supplementary Figure 2 – Aquileia, Roman theatre. General plan of the excavated trenches with location of the analyzed mortar samples.

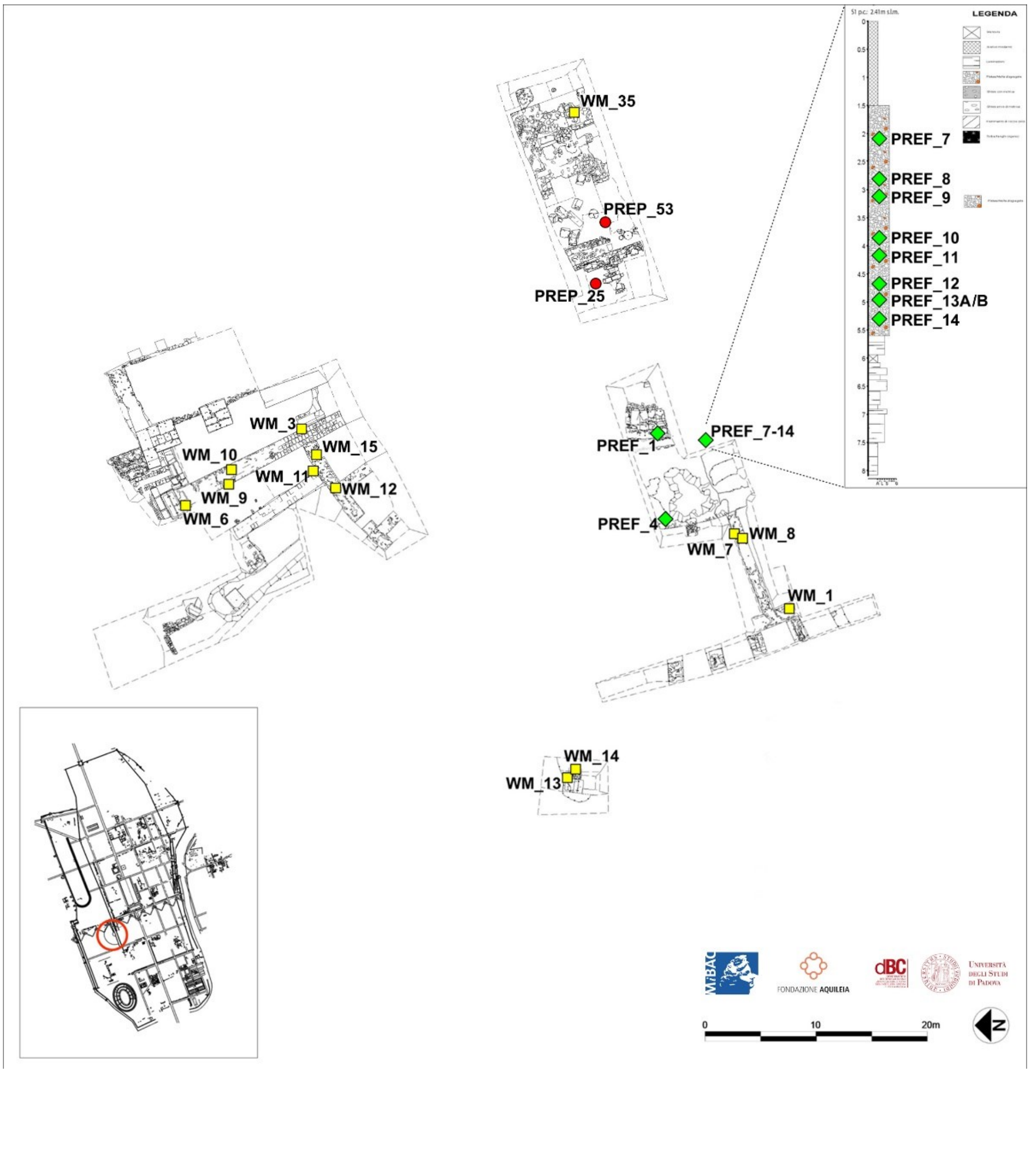

Supplementary Figure 3 - Sketch map of the Friuli Venezia Giulia region with indication of the main outcrop formations (author elaboration, based on [50], using Inkscape software v. 1.0.2 - https://inkscape.org/)

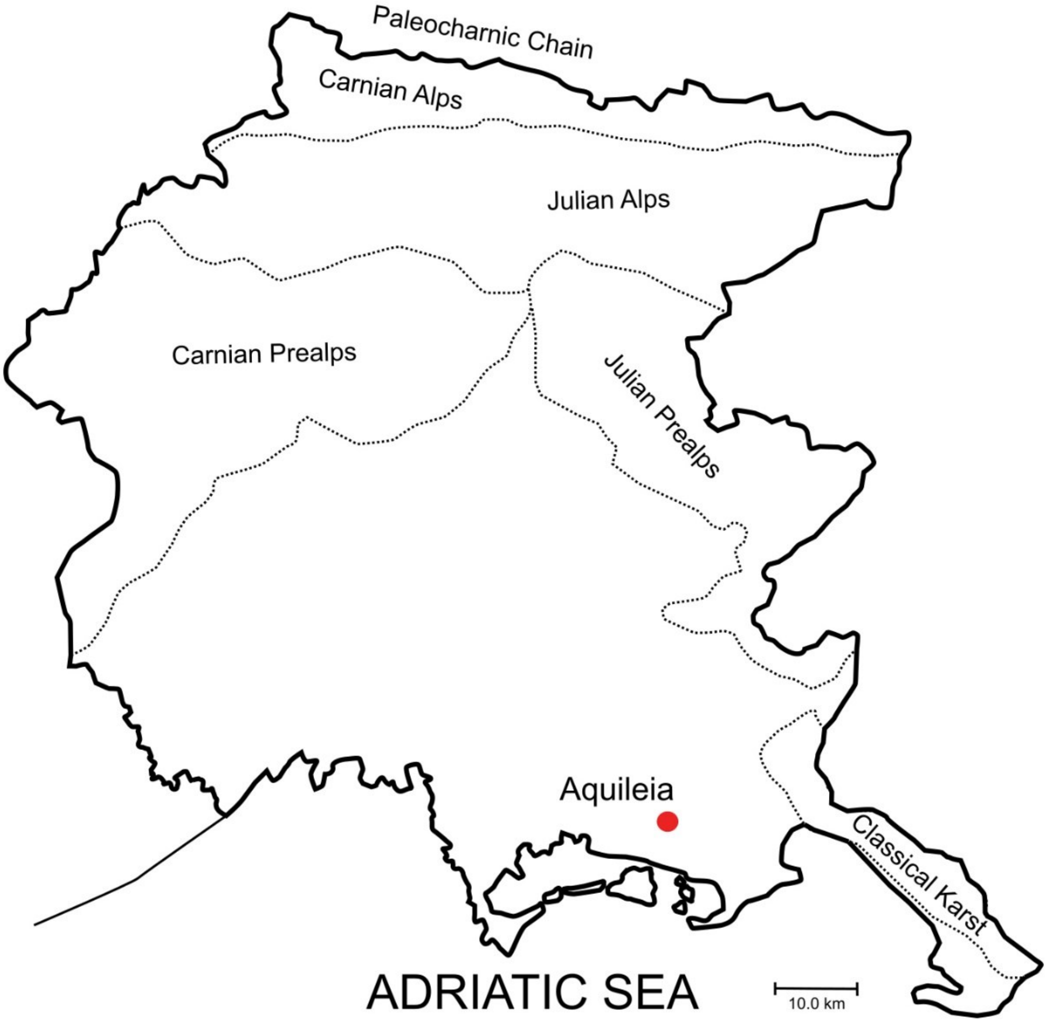

Aquileia is located in the lower plain of Friuli Venezia Giulia region (Italy), about 10 km from the coast. The regional orography is divided into different orographic units having a sedimentary origin and distributed from N to S in [49-50]:

(a) Paleocarnic Chain, consisting in Paleozoic limestone formations;

(b) The Carnian Alps and Julian Alps, characterized by Mesozoic formations with predominantly carbonate successions; i.e. dolomite and stratified dolomitic limestones (Dolomia Principale Formation), grey limestones (Dachstein Limestone Formation) and grey oolitic limestones (Calcari Grigi Formation - Lias);

(c) Carnian Prealps, overlooking the upper Friulian Plain, consisting of Mesozoic and Cenozoic carbonate rocks;

(e) The Julian Pre-Alps, constituting the Eastern continuation of the previous units and presenting Mesozoic and Cenozoic carbonate formations alternated with marly-arenaceous sandstones (flysch);

(f) The area of the Classical Karst, presenting different successions of carbonate rocks, from the Triassic to the Eocene, covered by flysch. In the Trieste Karst, successions from the Cretaceous to the Lower Eocene, consisting of prevalent bioclastic limestones (Karst limestones of Trieste, Aurisina Limestone) and, subordinately, dolomites.

Supplementary Figure 4 – Major elements variations vs SiO_2 of the volcanic clasts measured by SEM-EDS (average values) and XRF (tuff clast *w*).

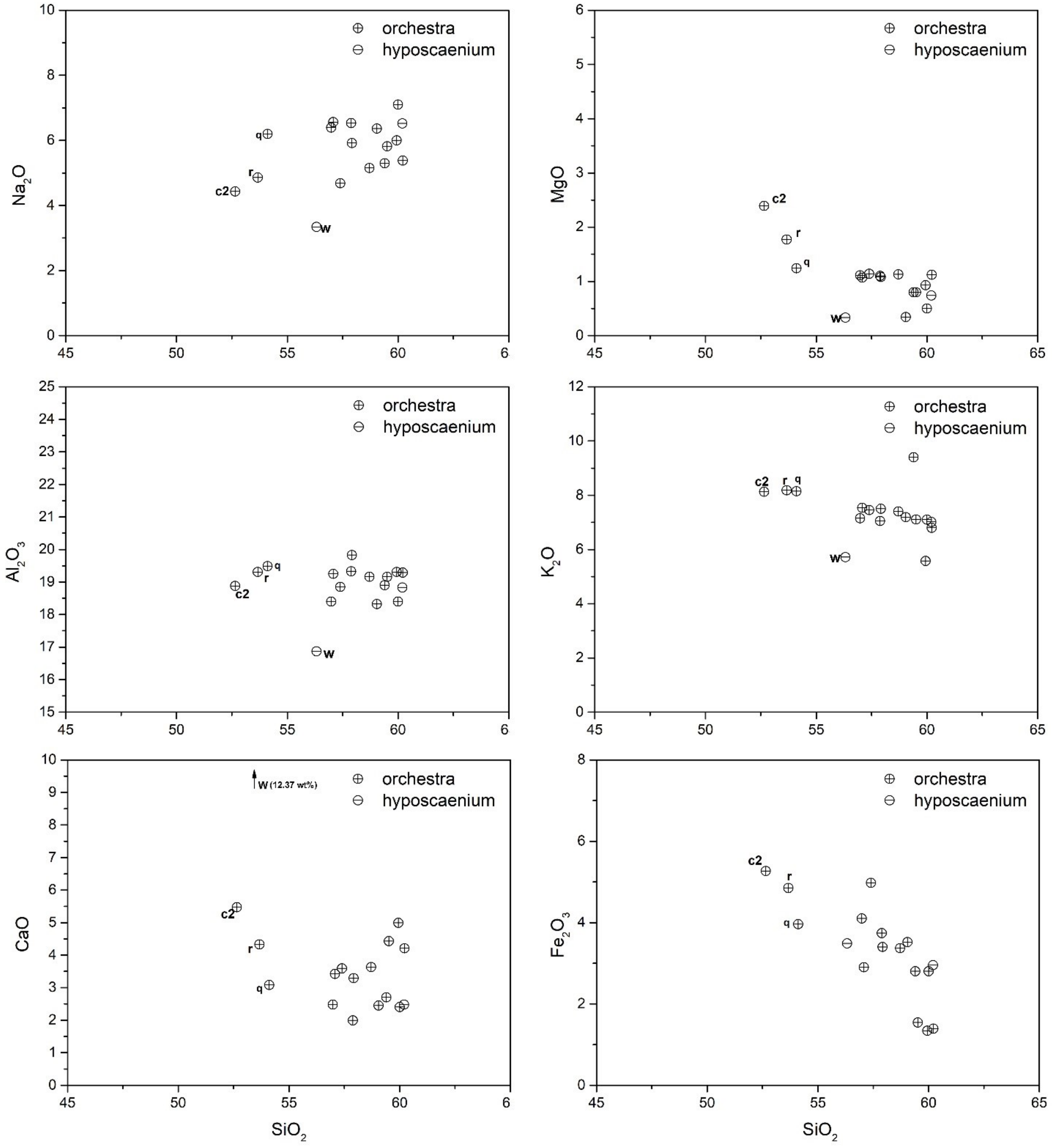

Supplementary Figure 5 – Spiderdiagram of trace elements (average values) of the volcanic glass measured by LA-ICP-MS. Data were normalized to the Primitive Mantle recalculated from pyrolite (PM, data from: McDonough, W.F., Sun, S.S., 1995. The composition of the Earth. Chem. Geol. 120, 223–253.)

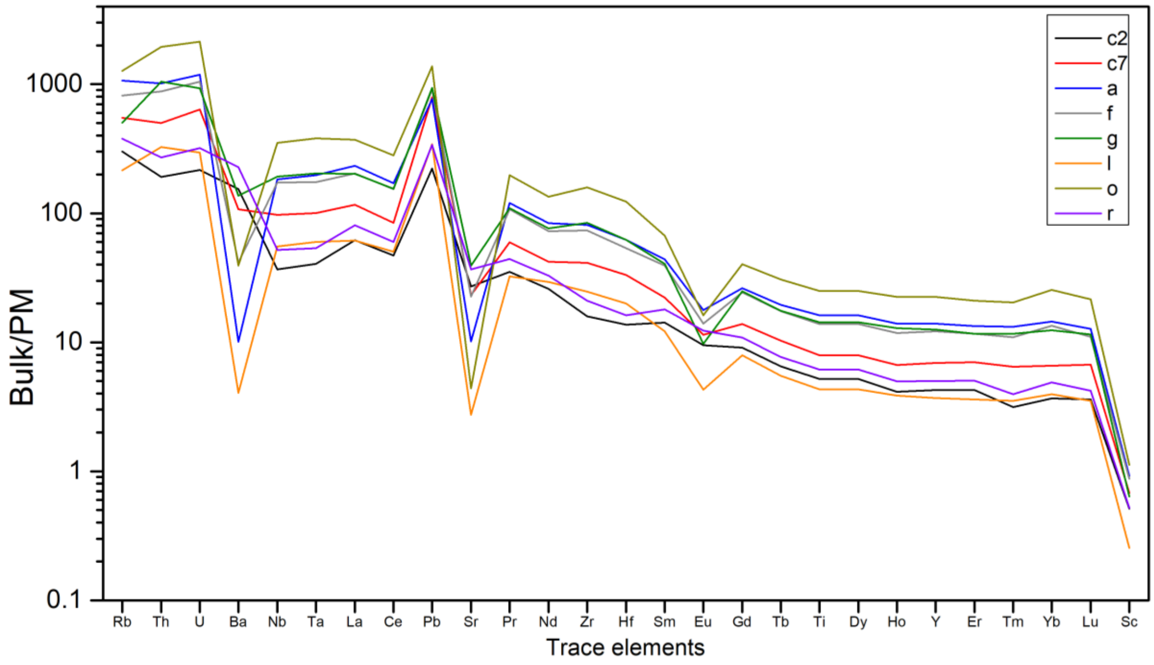

Supplementary Table 1 - Results of QPA-XRPD analysis of the binder fraction of a selection of mortar samples from the three groups, described as %wt. b.d.l. = Components below the detection limit.

Group	Sample	Calcite	M-S-H/Smectite	AFm	Quartz	Plagioclase	Diopside	Hematite	Muscovite	Amorphous
1	PREF_12	39.2	15.0	b.d.l.	0.7	b.d.l.	b.d.l.	b.d.l.	2.2	42.9
2	PREF_13B	43.5	8.1	2.4	0.5	4.5	18.6	0.6	b.d.l.	21.8
3	PREP_25.2	15.3	4.2	3.8	0.2	b.d.l.	2.3	0.1	b.d.l.	74.1

Supplementary Table 2 - Results of QPA-XRPD of the tuff clasts w in sample PREP_53. The phases not related with the original mineralogical profile of the clast are in italics.

Sample	Clast	Biotite	Ca-Plagioclase	K-Feldspar	Clinopyroxene	Chabazite	Phillipsite	Ilmenite	Smectite	Amorphous	*Vaterite*	*Calcite*	*Quartz*
PREP_53	w	1.6	8.3	12.4	5.8	3.4	11.1	0.4	7.9	40.7	*1.2*	*3.9*	*3.3*

Supplementary Table 3 - Geochemical composition of the major elements of the pumice clasts, analyzed by SEM-EDS, described as %Ox (mean values); b.d.l. = Components below the detection limit.

Sample	Clast	N° of measurments	Na2O		MgO		Al2O3		SiO2		P2O5		SO3		Cl2O		K2O		CaO		TiO2		MnO		Fe2O3	
			mean	*SD*	*mean*	*SD*	*mean*	*SD*	*mean*	*SD*	*mean*	*SD*	*mean*	*SD*	*mean*	*SD*	*mean*	*SD*	*mean*	*SD*	*mean*	*SD*	*mean*	*SD*	*mean*	*SD*
PREP_25	*c1*	6	5.30	0.12	0.80	0.19	18.90	0.11	59.40	0.65	b.d.l.	b.d.l.	b.d.l.	b.d.l.	0.50	0.06	9.40	0.33	2.70	0.01	0.20	0.12	b.d.l.	b.d.l.	2.80	0.10
PREP_25	*c2*	7	4.43	0.11	2.39	0.20	18.88	0.14	52.65	0.77	0.13	0.03	0.32	b.d.l.	0.69	0.04	8.13	0.23	5.47	0.02	1.26	0.13	0.59	0.15	5.27	0.19
PREP_25	*c3*	5	7.10	0.19	0.50	0.06	18.40	0.09	60.00	0.34	b.d.l.	b.d.l.	b.d.l.	b.d.l.	0.90	0.06	7.10	0.18	2.40	0.07	0.60	0.09	0.20	0.13	2.80	0.11
PREP_25	*c4*	8	6.39	0.38	1.11	0.10	18.40	0.86	56.98	0.85	b.d.l.	b.d.l.	0.15	0.12	0.75	0.20	7.15	0.25	2.48	0.40	1.40	0.25	1.10	0.16	4.10	0.39
PREP_25	*c5*	6	6.53	0.53	1.10	0.24	19.33	0.62	57.89	1.49	b.d.l.	b.d.l.	0.05	0.05	0.71	0.15	7.05	0.11	1.99	0.77	0.79	0.30	0.81	0.58	3.74	0.44
PREP_25	*c6*	6	6.36	0.88	0.34	0.42	18.32	0.25	59.05	0.41	0.17	0.13	b.d.l.	b.d.l.	0.92	0.30	7.19	0.46	2.45	0.35	1.07	0.31	0.62	0.45	3.52	0.64
PREP_25	*c7*	10	4.68	0.26	1.14	0.22	18.85	0.30	57.40	0.85	0.05	0.09	0.09	0.12	0.53	0.23	7.45	0.24	3.59	0.30	0.97	0.16	0.27	0.46	4.98	0.51
PREP_25	*a*	6	5.92	0.18	1.08	0.25	19.83	0.58	57.92	0.96	b.d.l.	b.d.l.	b.d.l.	b.d.l.	0.89	0.32	7.50	0.48	3.29	0.27	0.18	0.18	b.d.l.	b.d.l.	3.40	1.08
PREP_25	*e*	9	5.82	0.49	0.80	0.08	19.16	0.24	59.51	0.64	0.08	0.06	0.04	0.05	1.05	0.20	7.11	0.18	4.43	0.21	0.40	0.06	0.07	0.13	1.54	0.23
PREP_25	*f*	8	5.15	0.33	1.13	0.05	19.16	0.09	58.71	0.44	b.d.l.	b.d.l.	b.d.l.	b.d.l.	1.01	0.09	7.40	0.23	3.63	0.13	0.45	0.24	b.d.l.	b.d.l.	3.37	0.20
PREP_25	*g*	8	5.38	0.27	1.12	0.12	19.29	0.17	60.22	0.13	0.03	0.06	b.d.l.	b.d.l.	1.13	0.09	6.80	0.22	4.21	0.25	0.39	0.05	0.05	0.11	1.39	0.10
PREP_25	*l*	10	6.56	0.57	1.07	0.23	19.25	0.37	57.08	1.13	0.05	0.05	0.11	0.06	0.95	0.29	7.54	0.44	3.42	0.29	0.79	0.30	0.28	0.11	2.90	0.79
PREP_25	*o*	5	6.00	0.18	0.93	0.08	19.31	0.41	59.94	0.41	0.03	0.02	0.04	0.06	0.82	0.15	5.58	0.11	4.99	0.17	1.02	0.07	b.d.l.	b.d.l.	1.34	0.15
PREP_25	*q*	10	6.20	0.38	1.24	0.26	19.49	0.53	54.11	0.66	0.10	0.14	0.41	0.08	0.82	0.06	8.15	0.35	3.08	0.19	1.42	0.35	1.03	0.19	3.96	0.31
PREP_25	*r*	7	4.86	0.47	1.77	0.33	19.31	0.43	53.67	1.46	0.06	0.07	0.20	0.19	0.85	0.35	8.18	0.21	4.33	0.33	1.21	0.25	0.71	0.36	4.85	0.51
PREP_53	*z*	7	6.52	0.71	0.74	0.45	18.83	0.42	60.20	0.66	b.d.l.	b.d.l.	b.d.l.	b.d.l.	0.60	0.30	7.02	0.45	2.48	1.05	0.27	0.21	0.38	0.51	2.95	0.43

Supplementary Table 4 - Trace elements of the pumice clasts described as ppm, analyzed by LA-ICP-MS.

Clast	N° of measurments	Li	Be	Sc	V	Cr	Co	Ni	Cu	Zn	Rb	Sr	Y	Zr	Nb	Mo	Cs	Ba	La	Ce	Pr	Nd	Sm	Eu	Gd	Tb	Dy	Ho	Er	Tm	Yb	Lu	Hf	Ta	W	Pb	Th	U
c2	3	19	5.2	8.3	88	0.832	6.5	0.753	4.5	62	180	539	18	167	24	3.0	9.2	1013	40	79	9.0	33	5.8	1.47	5.0	0.643	3.5	0.615	1.86	0.214	1.62	0.243	3.9	1.5	3.16	33	15	4.4
c7	5	41	10	11	66	2.36	5.1	1.26	12	97	329	461	30	433	64	6.5	22	711	75	142	15	53	9.0	1.76	7.5	1.02	5.4	1.00	3.1	0.439	2.90	0.453	9.4	3.7	7.2	118	40	13
a	3	110	20	15	59	2.43	4.2	0.579	6.7	146	639	203	60	853	120	10	47	67	151	286	30	105	18	2.74	14	1.93	11	2.08	5.9	0.896	6.4	0.856	18	7.3	13	115	80	24
f	5	70	18	14	37	1.94	3.1	0.755	5.8	154	488	450	52	771	114	7.8	39	274	132	257	27	91	16	2.15	13	1.73	9.3	1.76	5.1	0.746	5.9	0.743	15	6.5	10	137	70	21
g	4	45	18	10	18	7.0	2.31	1.64	5.9	103	303	780	54	886	127	3.4	28	905	131	259	28	95	16	1.50	13	1.74	9.6	1.91	5.1	0.795	5.5	0.778	18	7.6	4.7	140	83	19
l	4	19	5.1	4.1	14	1.56	1.15	0.816	2.83	35	129	54	16	259	36	1.96	10	27	40	84	8.2	37	5.0	0.661	4.3	0.542	2.90	0.575	1.58	0.239	1.75	0.237	5.7	2.22	2.67	51	26	6.0
r	5	20	5.6	8.4	87	1.23	5.4	0.702	4.7	71	227	733	22	220	34	3.9	11	1498	52	101	11	41	7.3	1.90	5.9	0.763	4.2	0.743	2.21	0.268	2.15	0.284	4.6	1.99	4.2	51	22	6.5

Supplementary Table 5 - Major and trace elements of the tuff clast *w* (PREP_53) described as %Ox and ppm respectively, analyzed by XRF. b.d.l. = Components below the detection limit.

SiO_2	TiO_2	Al_2O_3	Fe_2O_3	MnO	MgO	CaO	Na_2O	K_2O	P_2O_5	Tot	L.O.I.
56.32	0.45	16.87	3.49	0.16	0.33	12.37	3.34	5.72	0.06	99.11	16.55

S	Sc	V	Cr	Co	Cu	Zn	Ga	Rb	Sr	Y	Zr	Nb	Ba	La	Ce	Nd	Pb	Th	U
356	23	35	12	b.d.l.	31	141	10	295	188	47	656	79	137	113	227	84	60	62	18

Supplementary Table 6 - NIST standards adopted to verify the EDS accuracy. In particular, SRM 2066 K411 is a specific reference material for SEM-EDX analysis.

Standard NIST-620 - Soda-Lime Flat Glass (NIST certificated data)

Element	wt%	Uncertainty
SiO_2	72.08	0.08
Na_2O	14.39	0.06
CaO	7.11	0.05
MgO	3.69	0.05
Al_2O_3	1.8	0.03
K_2O	0.41	0.03
SO_3	0.28	0.02
As_2O_3	0.056	0.003
Fe_2O_3	0.043	0.004
TiO_2	0.018	0.002

Results of EDS analysis of standard NIST-620 (mean of 5 spot analyses)

Element	mean (wt%)	SD
SiO_2	71.6	0.11
Na_2O	14.7	0.14
CaO	8.1	0.20
MgO	3.4	0.19
Al_2O_3	1.6	0.08
K_2O	0.5	0.01
SO_3	0.2	0.05

Standard NIST-2066-k411 - Glass microspheres (NIST certificated data)

Element	wt%	Uncertainty
Si	25.6	1.7
Ca	11.2	2.3
Mg	9.2	1.4
Fe	11.2	2.3
Oxygen	42.9	1.2

Results of EDS analysis of standard NIST-2066-k411 (mean of 5 spot analyses)

Element	mean (wt%)	SD
Si	24.7	0.15
Ca	11.7	0.09
Mg	9.7	0.10
Fe	10.3	0.13
Oxygen	43.6	0.07
